# Use of the Naturally Occurring Bacteriophage Grouping Model for the Design of Potent Therapeutic Cocktails

**DOI:** 10.3390/antibiotics13050385

**Published:** 2024-04-24

**Authors:** Tea Glonti, Michael Goossens, Christel Cochez, Sabrina Green, Sayali Gorivale, Jeroen Wagemans, Rob Lavigne, Jean-Paul Pirnay

**Affiliations:** 1 Laboratory for Molecular and Cellular Technology, Queen Astrid Military Hospital, B-1120 Brussels, Belgium; michael.goossens@mil.be (M.G.); christel.cochez@mil.be (C.C.); jean-paul.pirnay@mil.be (J.-P.P.); 2 Laboratory of Gene Technology, Department of Biosystems, KU Leuven, B-3001 Leuven, Belgium; sabrina.green@kuleuven.be (S.G.); sayali.gorivale@kuleuven.be (S.G.); jeroen.wagemans@kuleuven.be (J.W.); rob.lavigne@kuleuven.be (R.L.)

**Keywords:** phage cocktails, phage synergy, phage proto-cooperation, phage lytic activity, phage-resistant mutants, phage antagonism, inhibition by phage, phage isolation, phage Appelmans method, phage web pattern method

## Abstract

The specificity of phages and their ability to evolve and overcome bacterial resistance make them potentially useful as adjuncts in the treatment of antibiotic-resistant bacterial infections. The goal of this study was to mimic a natural grouping of phages of interest and to evaluate the nature of their proliferation dynamics with bacteria. We have, for the first time, transferred naturally occurring phage groups directly from their sources of isolation to in vitro and identified 13 *P. aeruginosa* and 11 *K. pneumoniae* phages of 18 different genera, whose host range was grouped as 1.2–17%, 28–48% and 60–87%, using a large collection of *P. aeruginosa* (n = 102) and *K. pneumoniae* (n = 155) strains carrying different virulence factors and phage binding receptors. We introduced the interpretation model curve for phage liquid culturing, which allows easy and quick analysis of bacterial and phage co-proliferation and growth of phage-resistant mutants (PRM) based on qualitative and partially quantitative evaluations. We assayed phage lytic activities both individually and in 14 different cocktails on planktonic bacterial cultures, including three resistotypes of *P. aeruginosa* (PAO1, PA14 and PA7) and seven *K. pneumoniae* strains of different capsular serotypes. Based on the results, the natural phage cocktails designed and tested in this study largely performed well and inhibited PRM growth either synergistically or in proto-cooperation. This study contributes to the knowledge of phage behavior in cocktails and the formulation of therapeutic phage preparations. The paper also provides a detailed description of the methods of working with phages.

## 1. Introduction

Developing control mechanisms to prevent the spread of antimicrobial resistance (AMR) is one of the greatest challenges of the modern world [1]. Each year, antibiotic-resistant bacteria cause more than 670,000 infections and 33,000 deaths [2]. *Pseudomonas aeruginosa* and *Klebsiella pneumoniae* are the pathogens most commonly associated with drug-resistant nosocomial infections. Carbapenem-resistant *P. aeruginosa* and *K. pneumoniae* carry resistance genes in a mobile genetic element that can be rapidly transferred between bacteria [3].

Therefore, there is a need to develop new treatments and approaches to control these bacteria. Bacteriophages, which have a narrow spectrum of activity, are considered to be very promising therapeutics in combination with antibiotics [4,5]. A phage cocktail expands the number of strains that the phages can infect and overcomes phage resistance that can occur when using single phages [1,6]. In recent years, successful in vitro and in vivo use of phage cocktails has been reported in the literature, including a variety of infections caused by *P. aeruginosa*, *K. pneumoniae*, *E. coli*, *A. baumannii* and *M. tuberculosis* [1,7,8,9,10,11]. It has often been shown that the phages are able to kill bacteria regardless of their Multidrug-resistant (MDR) phenotype [7]. These studies support the potential of phage cocktails in the control of MDR pathogens.

Fixed phage cocktails are likely to be the first bacteriophage therapeutics approved for widespread use in Western countries [4]. It is suggested that ready-to-use phage cocktails composed of a diverse genetic profile [7,12] be designed to support the improvement of host range and suppress the emergence of phage-resistant mutants (PRM). The host range of the phage defines the “depth and breadth” of the spectrum of activity of a phage cocktail [1,13]. Consequently, the optimal activity of phage cocktails could be predicted by considering host range overlap and could be used as screens to prevent PRM emergence and growth. Therefore, it is important to study phage host range, EOP (efficiency of plating) and lytic activity of phages in single and in cocktails in liquid to observe the mode of interplay of phages [1,10]. It is also very important to use a large panel of bacteria with different virulence factors, and MDRs are very important to design relevant cocktails to target other AMR bacterial strains to ensure their effectiveness against clinically significant pathogens. This was the primary goal of our study in the context of demonstrating whether naturally occurring phage groups (cocktails) have a broad spectrum of antimicrobial activity and to help differentiate the activity of individual phages from each other.

Although PRM growth is a major risk factor that must be considered and mitigated when planning the use of phage alone or with other antimicrobials to control bacterial infections, the emergence of PRMs could be beneficial in therapy, considering cases in which bacterial mutants with slow growth rates [14], minimal nutrient requirements and reduced antibiotic resistance are found. For example, resensitization to ceftazidime with evolved phage resistance was confirmed in the *P. aeruginosa* strain isolated from the fistula discharge [6]. Antibiotic resistance reduces bacterial virulence [6,14]. This is also an important consideration in favor of phage therapy.

This work aims to mimic the natural grouping of phages of interest and demonstrate the mode of their proliferation dynamics with bacteria: synergy, proto-cooperation, or antagonism. Moreover, we introduced the interpretation model curve for phage liquid culturing, which allows easy and quick analysis of bacterial and phage co-proliferation and growth of phage-resistant mutants (PRM) based on qualitative and partially quantitative evaluations. The data demonstrated the advantage of phage bi- and tri-cocktails composed of the different genera over single phages in controlling PRM growth in vitro, and we found that phages are better “trained” in natural river water than in urban/hospital sewage water. The approach of using a bacterial panel of relevant diversity to assess phage virulence with respect to antibiotic-resistant strains and PRM emergence was again shown to facilitate the evaluation and differentiation of the therapeutic potential of individual phages and phage cocktails. In addition, the paper gives a detailed description of methods for working with phages.

## 2. Results

### 2.1. Isolation of Phages

**Figure 1:**
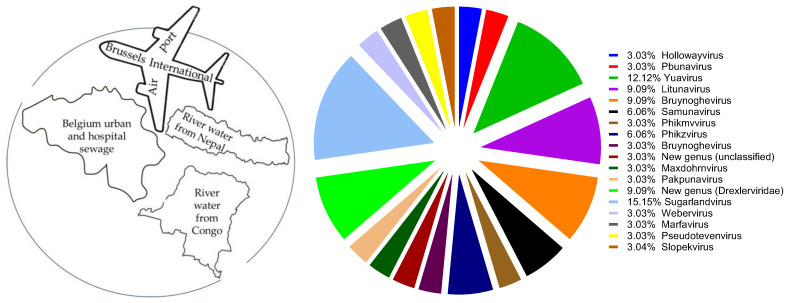
Sources of phage isolation (**left**) and distribution of newly isolated phages to different genera (**right**).

A total of 13 *P. aeruginosa* and 11 *K. pneumoniae* phages were newly isolated from urban (the international terminal of Brussels (Zaventem airport)) and hospital sewage and environmental waters (Figure 1 and Table 1 and Table 2). The bacterial matrix included 102 *P. aeruginosa* strains with different genetic backgrounds and 155 *K. pneumoniae* strains representing 36 *Klebsiella* capsular serotypes (Tables S1 and S2). In total, the newly isolated phages belonged to 18 different genera, according to genome analysis.

### 2.2. Phage Plaque Purification and Morphology Characterization

Plaque purification of the newly isolated phages was performed on their isolating host strains (PAO1K, CN573 and PAV237 for *P. aeruginosa* and LabMCT0682, ATCC 27736, NCTC 13438 and SB4385 for *K. pneumoniae*), which is important to maintain a standardized manner of morphological differentiation of characteristics of plaques in each round of passages and to minimize phage mutation as much as possible. Phage plaque diameters in soft (0.7%) agar ranged from 1 to 7 mm. The detailed description of the phage plaque morphology, genome size, bacterial strains of isolation and their reference numbers are given in Table 1 and Table 2.

One *P. aeruginosa* phage (Atpa007) and four *K. pneumoniae* phages (Atkp002, Atkp003, Atkp011 and Atkp013) were excluded from further experiments as they did not give reproducible results after the plaque purification steps.

### 2.3. Phage Host Range Study

**Figure 2:**
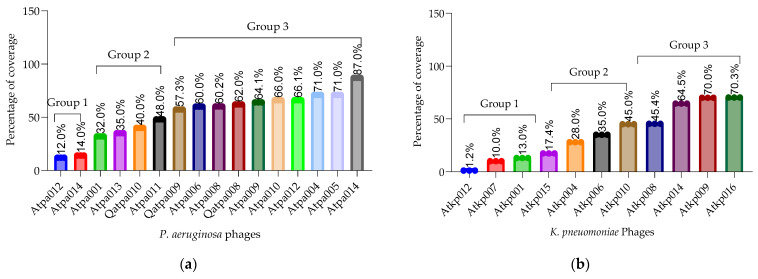
(**a**) *P. aeruginosa* host range (%); (**b**) *K. pneumoniae* host range (%).

Plaque-purified phages were propagated using the web pattern plate method to generate the phage stock lysates for downstream experiments. Host range and efficiency of plating (EOP) were determined in triplicate using a large collection of *P. aeruginosa* (n = 102) and *K. pneumoniae* (n = 155) strains carrying different virulence factors and phage binding receptors. Figure 2 shows that the host coverage of *P. aeruginosa* is in the range of 12–87% (including three different resistotypes PAO1, PA14 and MDR PA7) [15,16,17,18] and K. pneumonia is in the range of 1.2–70.03% (including 36 *Klebsiella* capsular serotypes). *P. aeruginosa* phage Atpa014 and *K. pneumoniae* phage Atkp016 showed the highest host range.

Figure 3 (each violon plot) shows the average of each phage EOP (efficiency of plating) on the tested bacterial panel (*P. aeruginosa* (n = 102) and *K. pneumoniae* (n = 155)). The EOP value is in the range of 0.0005–1.9 for *P. aeruginosa* and 0.01–1.0 for *K. pneumoniae* phages. Figure 3 shows only the phages isolated in this study. The relevant EOP results (the ratio of the titer (cfu/mL) produced on a test bacterial strain to the phage titer of a propagating bacterial host strain) for all *P. aeruginosa* and *K. pneumoniae* phages, separately, are given in the Supplementary Materials (Figures S1–S25).

### 2.4. Appelmans Assay

**Figure 4:**
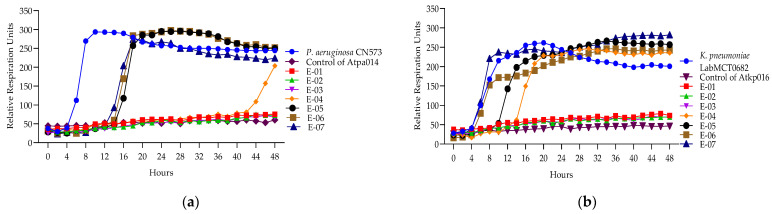
(**a**) Appelmans assay of *P. aeruginosa* phage Atpa014; (**b**) Appelmans assay of *K. pneumonia* phage Atkp016.

**Figure 5:**
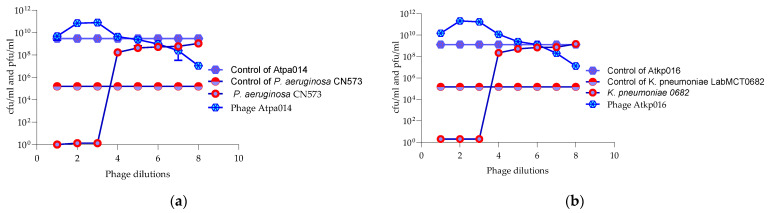
Enumeration of bacteria (cfu/mL) and phages (pfu/mL) after 24 h of incubation (Appelmans assay). (**a**) *P. aeruginosa* strain CN573 and phage Atpa014. (**b**) *K. pneumoniae* strain LabMCT0682 and phage Atkp016.

The Appelmans assay was performed in triplicate on the 16 *P. aeruginosa* 11 *K. pneumoniae* phages (Figures S26–S50). Figure 4 shows only the proliferation of *P. aeruginosa* phage Atpa014 proliferation on strain CN573 and phage *K. pneumoniae* phage Atkp016 on strain LabMCT0682, as these phages almost repeat the “classic picture” of Appelmans’ dilutions (clear–less; clear–turbid). Most of the phages had a clearing effect (cleared well content with no colony-forming cells) at phage −bacteria (P:B) ratios of 10, 100 and 1000. However, clearing does not mean that the phage has reached its maximum capacity in that particular case, and conversely, turbidity does not mean that the phage has not been replicated. Therefore, all test samples were subjected to bacterial and phage enumeration tests after 24 h of incubation in the OmniLog system. The results confirmed that the bacterial culture sampled in clear wells with up to 60 rru (relative respiration units) was not reproducible and showed that most of the phages again gave the highest titers at P:B of 100 and 1000 (dilutions E-02 and E-03) (Figure 5).

### 2.5. Lytic Activity of Phages and Phage Cocktails

#### 2.5.1. Design of Phage Cocktails

The lytic activity study included single phages and phage cocktails. Seven different cocktails were assembled for both *P. aeruginosa* and *K. pneumoniae* phages.

The cocktails were largely designed based on the phage isolation sources (Table 3 and Table 4) to mimic the natural grouping of the phages. However, due to the loss of some phages that were considered to be temperate ones, several cocktails were assembled (in bi- or tri-cocktails where possible) according to the phages’ EOPs, Appelmans results and tracking a diversity of phage receptors. Based on the Appelmans results, a P:B ratio of 100 was selected from two (1:100, 1:1000) to be used in lytic activity studies for all phages in the interest of standardization. The cocktails were developed by mixing the same volume and pfu/mL of each phage component, and the total pfu/mL was considered in the activity test to achieve a P:B ratio of 100.

#### 2.5.2. Selection of Bacterial Strains for Testing the Activity of Single Phages and Cocktails

The evaluation of the lytic activity of single phages and cocktails was performed on a total of 16 strains; in particular, 8 *P. aeruginosa* strains with different virulence (PAO1K, CN573, PAV237, A11, Is573 and Is580, including three resistotypes of PAO1, PA14 and PA7) and seven *K. pneumoniae* strains (LabMCT0682, atcc 2736, nctc 13438, 10394, VPKP389, SB4385, 70165) of the different capsular serotypes, as we aimed to evaluate the extent of lytic activity of the cocktails in terms of host range to address MDR/virulent strains and to differentiate them from each other. The virulence characteristics/capsular types of these strains and their lytic activity are shown in Table 5 and Table 6.

#### 2.5.3. Developing a Model to Interpret Bacterial Proliferation and Phage Activity Curves

**Figure 6:**
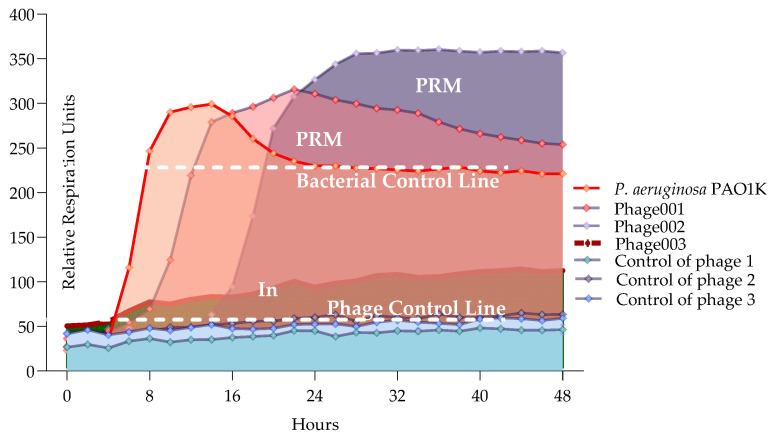
Interpreting model for phage and bacteria proliferation using all PLC.

Here, we developed the interpretation model curve for PLC (phage liquid culturing) experiments (Figure 6). OmniLog Data Analysis Software (v1.7) was used for data collection and visualization of kinetic assay results. The interpretation of the generated bacterial proliferation curves was performed as follows (Figure 6): 1. A relative respiration unit (rru) in the range of 0–60 was considered to be as indicative of the phage clearing (CL) effect (absolute lysis) based on the experimental results averaged for phage controls. 2. Bacteria–phage growth curve that did not have the same shape as the control bacterial growth curve and resulted in a higher rru was considered as phage-resistant mutant (PRM) growth. 3. Bacterial proliferation curve with shorter exponential phase and reduced and constant rru maintained up to 48 h compared to bacterial control was evaluated as an indication of bacterial inhibition (In) effect by phage.

The type of interaction between phage components in a cocktail, where the combination of phages produces a more potent effect over the course of the experiment than the individual phages, was evaluated as synergistic activity.

The type of interaction between phage components in a cocktail, where the combination of phages produces the same potent effect as the individual phages, and they do not need to interact with each other to enhance the effect over the course of the experiment, was evaluated as proto-cooperation.

The type of interaction between phage components in a cocktail, where the combination of phages produces a less potent effect than the individual phages over the course of the experiment, was evaluated as antagonism.

#### 2.5.4. Lytic Activity of Individual Phages and Phage Cocktails

The results of phage and cocktail lytic activities are shown in Figure 7, Figure 8, Figure 9, Figure 10, Figure 11, Figure 12, Figure 13 and Figure 14 and Figures S51–S57. The evaluation of the types of interactions of the phage components in the cocktails is given in Table 5 and Table 6. All PLC experiments were performed in at least triplicate.

**Figure 7:**
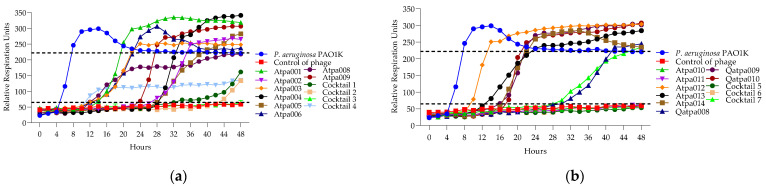
(**a**) Lytic activity curves of *P. aeruginosa* phage’s Atpa001–Atpa009 and cocktails 1–4 on PAO1K; (**b**) lytic curves of *P. aeruginosa* phage’s Atpa009–Atpa014, Qatpa008–Qatpa010 and cocktails 5–7 on PAO1K.

Looking at Figure 7, in the case of cocktails 1, 2, 3 and 6 on *P. aeruginosa* PAO1K (used mainly as a propagation strain), we see the prolongation of the clearing (absolute lysis) time up to 32 h, 42 h, 48 h, respectively, and the relatively reduced rru at the endpoint when comparing the individual phage components, which mostly show PRM (phage-resistant mutant) growth. For example, in the case of cocktail 1, the clearing effect is maintained for 32 h (in the case of individual phages, it is for 12–24 h), and at the endpoint of 48 h the rru is reduced to 161 (in the case of individual phages, rru is 248, 265, 319, respectively). Accordingly, the interaction between individual phages in cocktails of *P. aeruginosa* (1, 2, 3 and 6) on PAO1K was evaluated as synergistic (Table 5). Cocktails 5 and 7 showed the same clearing time for 48 h and 30 h, respectively, as all or at least one of the individual phage components. For example, the clearing time (30 h) of cocktail 7 and a phage component of Qapta008 is the same, but the rru is reduced in the case of the cocktail (Figure 7). Based on this, the interaction between the individual phages in cocktails 5 and 7 was evaluated as a proto-cooperation (Table 5). Cocktail 4 showed inhibition of PAO1K growth for 48 h (constant reduction in rru to 135 compared to the control curve rru of 221). In this case, however, the interaction between the phage components was evaluated as antagonism since the individual phage components (Atpa008 and Atpa009) showed a longer clearing effect (for 10 h and 24 h, respectively) compared to cocktail 4.conclusion, we propose the following

**Figure 8:**
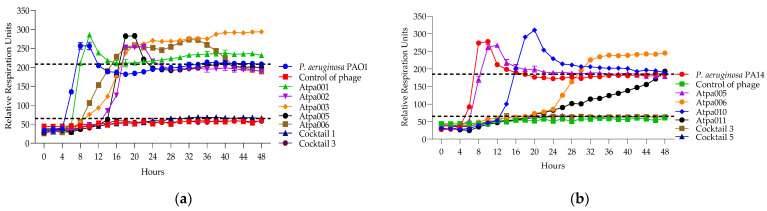
(**a**) The lytic activity curves of *P. aeruginosa* phage’s Atpa001–Atpa006 and cocktails 1, 3 on PAO1; (**b**) the lytic activity curves of *P. aeruginosa* phage’s Atpa005–Atpa006 and Atpa010–Atpa011 and cocktails 3, 5 on PA14.

Figure 8 shows that only cocktails 1, 3 and 3, 5 are active on PAO1 and PA14 strains, developing synergistic interaction. And in the case of the individual phages of Atpa003 and Atpa006, PRMs are grown.

**Figure 9:**
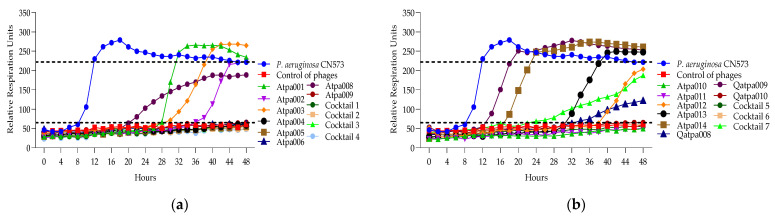
(**a**) Lytic activity curves of *P. aeruginosa* phage’s Atpa001–Atpa009 and cocktails 1–4 on CN573; (**b**) lytic activity curves of *P. aeruginosa* phage’s Atpa009–Atpa014, Qatpa008–Qatpa010 and cocktails 5–7 on CN573.

In Figure 9, we can see that cocktails 1 and 6 are active on *P. aeruginosa* CN573 for a longer time of 48 h than the individual phage components (synergy), while cocktails 2–5 show proto-cooperation as there is no improved effect after the experimental time of 48 h compared to the individual phage components or at least one of them. The individual phage proliferation curves of Atpa003, Atpa013 and Qatpa009 show the growth of PRMs.

**Figure 10:**
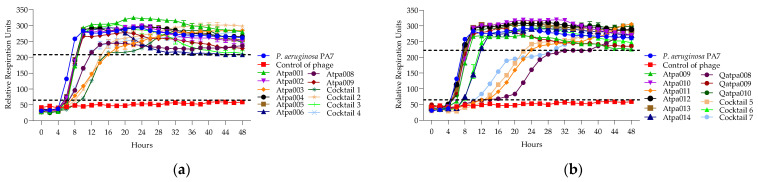
(**a**) The lytic activity curves of *P. aeruginosa* phage’s Atpa001–Atpa009 and cocktails 1–4 cocktails 5–7 on PA7; (**b**) the lytic activity curves of *P. aeruginosa* phage’s tpa009–Atpa014, Qatpa008–Qatpa010 and cocktails 6–7 on PA7.

In Figure 10, we can see that cocktail 1 shows synergistic activity; cocktails 5 and 6—proto-cooperation; and cocktails 2, 4 and 7—antagonistic activity on *P. aeruginosa* strain PA7. The individual phage proliferation curves of Atpa003, Atpa013, Atpa010, Atpa011 (after 32 h) and Qatpa008 (after 40 h) show the growth of PRMs.

**Figure 11:**
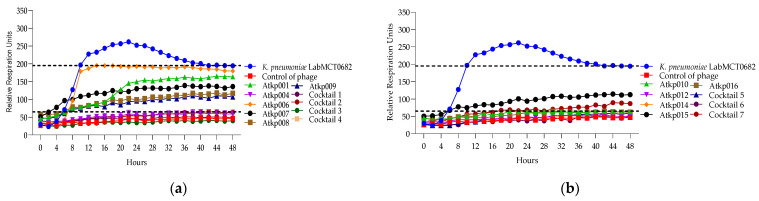
(**a**) Lytic activity curves of *K. pneumoniae* phage’s Atkp001–Atkp009 and cocktails 1–4 on LabMCT0682; (**b**) lytic activity curves of *K. pneumoniae* phage’s Atkp009–Atp016 on LabMCT0682.

In Figure 11, we can see that *K. pneumoniae* phage Atkp004, Atkp012 and Atkp016 give a clearing effect, and the rest of Atkp001, Atkp006, Atkp007, Atkp008, Atkp009, Atkp010 and Atkp015 phages give inhibitory effect individually, but in all cocktails, they develop synergy (cocktails 1, 2 and 4) and proto-cooperation (cocktails 3, 5, 6 and 7) (Table 6).

**Figure 12:**
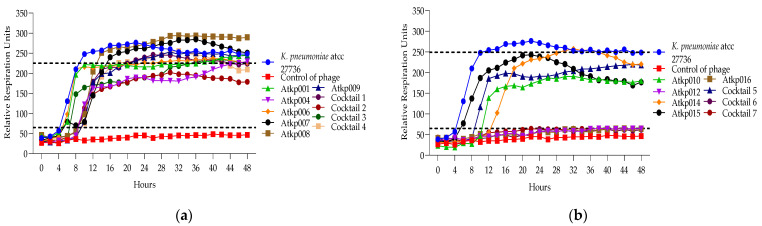
(**a**) Lytic activity curves of *K. pneumoniae* phage’s Atkp001–Atkp009 and cocktails 1–4 on atcc 27736; (**b**) lytic activity curves of *K. pneumoniae* phage’s Atkp009–Atp016 and cocktails 5–7 on atcc 27736.

In Figure 12, we can see that *K. pneumoniae* phage Atkp001 and Atkp009 do not individually give clearing effects, but in cocktail 2, they show clearing not for a long time but for 8 h; therefore, it was evaluated as synergistic activity. Cocktails 6 and 7 show proto-cooperation.

**Figure 13:**
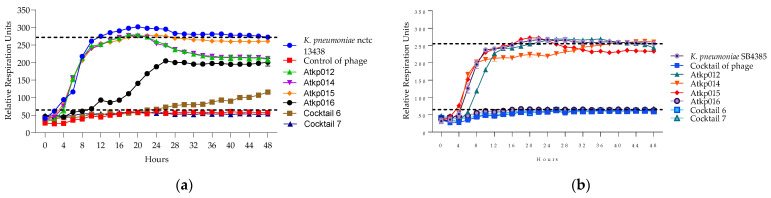
(**a**) Lytic activity curves of *K. pneumoniae* phage’s Atkp012–Atkp016 and cocktails 6–7 on nctc 13438; (**b**) lytic activity curves of *K. pneumoniae* Atkp012–Atkp016 and cocktails 6–7 on SB4385.

In Figure 13, we see a clear picture of the synergy of cocktails 6 and 7 on the different strains of *K. pneumoniae* nctc 13438 and SB4385 as they prolong the clearing effect for 48 h compared to the single phage component of Atkp016, where the clearing effect lasts for 16 h.

**Figure 14:**
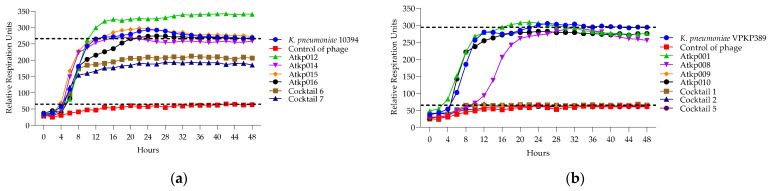
(**a**) Lytic activity curves of *K. pneumoniae* phage’s Atkp012–Atkp016 and cocktails 6–7 on 10394; (**b**) lytic activity curves of *K. pneumoniae* Atkp012–Atkp016 and cocktails 6–7 on VKPKP389.

In Figure 14a, we see that the single phage components Atkp012, Atkp014, Atkp015 and Atkp016 do not give any clearing effect on *K. pneumoniae* strain 10394, but in cocktails 6 and 7, they show an inhibitory effect with reduced rru of 206 and 185, respectively, compared to the control bacterial rru of 265. In Figure 14b, we see a clear synergistic activity of cocktail 1 and 2 on *K. pneumoniae* strain VKPKP389, as cocktail 1 prolongs the clearing effect from 12 h to 48 h, and cocktail 2 gives clearing effect for 28 h, while the component phage Atkp001 does not show any clearing and Atkp009 gives inhibitory effect for 48 h.

#### 2.5.5. Bacteria and Phage Enumeration

**Figure 15:**
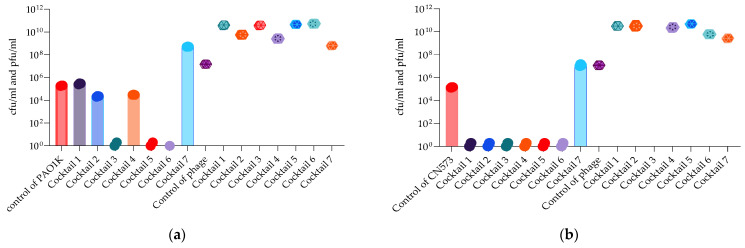
Bacterial (cfu/mL) and phage (pfu/mL) counts after 48 h of OmniLog incubation for 7 *P. aeruginosa* phage cocktails and strains: (**a**) PAO1K; (**b**) CN573. Bars with the spere symbols represent cfu/mL, and the hexagonal symbols without bars represent pfu/mL.

**Figure 16:**
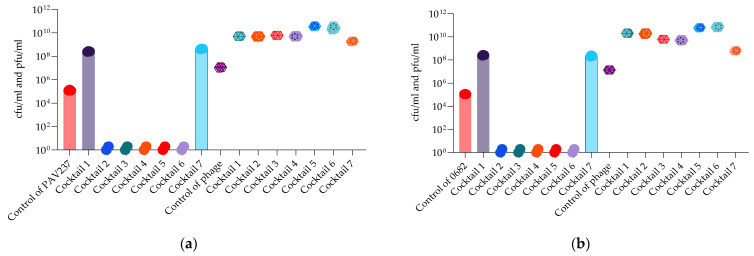
Bacterial (cfu/mL) and phage (pfu/mL) counts after 48 h of OmniLog incubation for 7 *P. aeruginosa* phage cocktails and strains: (**a**) PAV237; (**b**) *K. pneumoniae* phages and strain LabMCT0682. Bars with the spere symbols represent cfu/mL, and the hexagonal symbols without bars represent pfu/mL.

Bacterial and phage enumeration assays were performed on the phage propagating host strains after 48 h of incubation (endpoint) in the OmniLog system. The purpose of this study was to confirm that phage and cocktail activity in the liquid (OmniLog System) was correlated with the results obtained on agar (MD/SP DAL method). Figure 15 and Figure 16 show that three *P. aeruginosa* phage cocktails (3, 5, 6) lyse PAO1K, while almost all cocktails (except 7) lyse CN573 and five cocktails (2, 3, 4, 5, 6) lyse PAV237. For *K. pneumoniae*, five cocktails (2, 3, 4, 5, 6) lyse LabMCT0682.

## 3. Discussion

In this study, we isolated 13 *P. aeruginosa* and 11 *K. pneumoniae* phages and evaluated the lytic activity of the natural grouping of *P. aeruginosa* and *K. pneumoniae* phages on planktonic bacterial cultures. Collectively, the results show that phages isolated from the same environmental source (urban and hospital sewage or rivers) can be quite different. Based on genome sequence analysis, 6/7 phage cocktails in *P. aeruginosa* were composed of phages from different genera, while 3/7 phage cocktails in *K. pneumoniae* were composed of phages from different genera (Figure 1, Table 1). This mixed bouquet of phages is the result of our isolation approach using large panels of host bacteria with different genetic backgrounds and geographical origins.

According to the results of the EOP, the phages of both species could be divided into three groups based on the percentage of the host coverage: (1) 10–17%, (2) 28–48% and (3) 60–87% (Figure 2). In general, *P. aeruginosa* phages have a slightly broader host range than *K. pneumoniae* phages. However, on average, *K. pneumoniae* phages have a higher EOP (one log less reduction in the test bacterial strain) than *P. aeruginosa* phages. It is important to emphasize that the phage isolated and studied here were not trained or adapted to the different strains to extend the host range and EOP, as we wanted to preserve them as wild. However, *P. aeruginosa* phages Atpa014 and Qatpa008 and *K. pneumoniae* phage Atkp010 have relatively high average EOPs of 1.9, 7.8 and 1.02, respectively. But if we compare them with the lytic activity in cocktails, we do not see the advantage because phage cocktails with lower average EOP show better performance in PLC experiments.

The interpretation model developed in this study (Figure 6) allows for easy and quick analysis of bacterial and phage co-proliferation based on qualitative (changes in the shape of different phases of the bacterial growth curve) and partially quantitative (changes in relative respiration units (rru)) evaluations. Specifically, a bacterial culture in a favorable and stable condition typically goes through four phases (lag, exponential, stationary and death) and produces a constant curve shape, whereas a bacterial culture with phage(s) either repeats the same growth curve shape or differs from it. Using the OmniLog system, which generates high-throughput kinetic readouts based on the metabolic activity of bacterial cells, we were able to directly compare these differences and assess how the phages (at different P:B ratios and in different combinations) affect bacterial growth and classify the bacteria–phage relationship as synergistic, proto-cooperation or antagonistic.

Phage virulence, the emergence of PRMs and their correlation with bacterial virulence and antibiotic resistance are of great importance for the therapeutic use of phages. Therefore, we used a very diverse bacterial panel (including three different resistotypes of *P. aeruginosa* PAO1, PA14 and MDR PA7: PAO1 and PA14 are characterized by possessing the T3SS (type III secretion system) and the corresponding effector toxins (exoS and exoU, respectively), whereas PA7 uses the TPS (two-partner secretion system), secreting the ExlA exolysin to the damaged surrounding tissue cells [19]; the exoU genes are correlated with resistance to aminoglycosides and fluoroquinolones [20,21]) and six *K. pneumoniae* strains with different capsular serotypes of K1, K2, K27, K30, K62, K81 and one strain ST258 producing the KPC-3 carbapenemase (nctc 13438).

Overall, our interpretation of the PLC results (Table 5 and Table 6) shows that both *P. aeruginosa* and *K. pneumoniae* phage cocktails have a greater lytic effect over 48 h than their individual phage components. They showed either synergistic activity when the combination of phages gives a higher potent effect expressed in a prolonged clearing (absolute lysis) over 48 h, or proto-cooperation when the combination of phages gives the same potent effect as in single mode, and they do not need to interact with each other to improve clearance over 48 h. The propagating strains of *P. aeruginosa* (PAO1K, CN573 and PAV237) were lysed by 6/7 cocktails, while the propagating strain of *K. pneumoniae* (LabMCT0682) was lysed by all 7 cocktails. For non-propagating strains, mostly two cocktails of each species showed lytic activity for 48 h, with some exceptions; *P. aeruginosa* PA7 was cleared by three cocktails (1, 5, 6), but for 10 h, 14 h and 8 h, respectively; *K. pneumoniae* strain 10394 of capsular serotype 62 was cleared by cocktail 4 for 10 h; strain 70165 of capsular serotype 2 was not cleared by any phage/cocktails, only cocktails 6 and 7 showed the inhibitory effect, and it was evaluated as synergy as rru in the case for both cocktails were reduced by 48 h compared to the component phages.

We observed that individual *P. aeruginosa* phages induced more PRM (phage-resistant mutant) growth than *K. pneumoniae* (Table 5 and Table 6), and the reason for this may be that *P. aeruginosa* generally grows faster [22] than *K. pneumoniae*. This assumption is supported by the fact that all phages show PRM growth on the PAO1K propagating strain, i.e., they multiply faster on the adapted strain, and consequently, the PRM emergence rate is increased. In addition to that, we identified several antagonistic activities only in the case of *P. aeruginosa* cocktails, in particular, cocktail 4 on PAO1K; cocktail 7 on CN573 and PAV237; and cocktails 2, 4 and 7 on PA7. In all cases, the antagonism is accompanied by PRM growth. Based on this, we can assume that antagonism is somehow related to PRM growth, as we did not observe it in cases of *K. pneumoniae* cocktails.

Using the bacterial panels of bacterial strains with the different virulence factors, we can conclude that *P. aeruginosa* and *K. pneumoniae* cocktails have overall very diverse infection patterns, but *K. pneumoniae* cocktails show a less diverse lytic effect pattern compared to *P. aeruginosa* (Table 5 and Table 6).

Furthermore, no correlation was observed between the virulence factor or capsular serotype of the bacterial strains and the activity of the cocktails. For example, *P. aeruginosa* strains PAO1 (O5-serotype, T3SS, ExoS, ExoY), PA14 (O10-serotype, T3SS, ExoU, ExoY) and Is573 (O11-serotype, ExoU) with the different virulence factors are lysed by the different phage cocktails of 1, 5 and cocktails 6 and 7, respectively. Strain PAO1 [23] is lysed by two cocktails of 1 and 3, while PAO1K [24] is lysed by all seven cocktails (Table 5). Four out of seven *K. pneumoniae* strains belonging to the different capsular serotypes of 30, KPC-3, K27 and K2 are inactivated for 48 h by the same cocktails of 6 and 7 (Table 6). This fact allows us to conclude that the cocktails have broad lytic activity in terms of bacterial virulence.

Some *K. pneumoniae* cocktails in PLC showed the bacterial inhibition effect; this occurs when the exponential phase of the bacteria–phage growth curve plot is shorter, and the rru is constant and reduced compared to those for the bacterium alone. We did not consider this phenomenon as PRM growth, which would result in a more skewed shape of the exponential slope, resulting in a delayed plateau due to the slow growth of PRM [14]. There is no correlation between the capsule type and this inhibitory effect, e.g., strains belonging to capsular serotypes K30, K62 and K2 are inhibited by cocktails 2, 6 and 7 and 2, 3 and 5, respectively (Table 6). In these cases, the inhibitory effect was evaluated as a synergistic activity, as the cocktails resulted in a reduction in rru at the endpoint (48 h) compared to the individual components (Table 6). Certainly, the inhibiting effect cannot be considered a perfect option like clearing, but it still results in a reduction in infection units. Moreover, considering that this type of event occurs in parallel with the PRM growth in vivo treatment process, which triggers resensitization [6,14], it could eventually have a potential clinical effect. In the case of *P. aeruginosa*, cocktail 4 showed an inhibitory effect on the propagating host strain of PAO1K. However, the inhibition was evaluated as an antagonistic activity as the individual phage components resulted in a clearing effect for quite some time; e.g., the phage Atpa009 alone resulted in a clearing effect for 20 h, in contrast to cocktail 4, which resulted in a constant inhibitory effect for 48 h (Table 5).

Finally, we found “the interpretation model curve for phage liquid culturing” developed here to be very useful and timeless in analyzing/evaluating bacterial and phage co-proliferation and growth of phage-resistant mutants PRM in liquid. The most promising cocktails were *P. aeruginosa* cocktails 3 (urban source) and 5 (river water from Nepal) and *K. pneumoniae* cocktails 6 (river water from Nepal and Congo) and 7 (river water from Congo) (Table 5 and Table 6). All of these cocktails are bi-cocktails composed of the different phage genera, and three of them were isolated from rivers, suggesting that phages are better ”trained” in natural river water than in urban/hospital sewage water. In addition, the use of a relevant bacterial panel to assess in vitro or in vivo phage virulence and PRM emergence facilitates the evaluation and differentiation of the therapeutic potential of individual phages and phage cocktails.

In the future, we plan to perform deep genomic analyses of the newly isolated phages presented in this study to better characterize each phage in terms of therapeutic safety issues and to better understand phage–bacteria interaction mechanisms during their co-proliferation, particularly the inhibitory effect by phage, antagonism and their correlation with PRM emergence. Furthermore, our goal is to test the selected phages and relevant MDR strains with the different virulence factors in vivo using a *Galleria mellonella* larvae infection model, to interpret and evaluate all in vitro and in vivo data and to predict/translate them to support the therapeutic use of the tested phages.

In conclusion, we propose the following:

- The natural groups (cocktails) of phages isolated from the same environmental source (urban and hospital sewage or rivers) are quite different. For *P. aeruginosa*, 6/7 phage cocktails were composed of phages belonging to different genera, while for *K. pneumoniae*, this was the case for 3/7 phage cocktails.
- The natural groups (cocktails) of phages have relatively broad host range, as at least two of them showed in vitro clearing effect on *P. aeruginosa* strains of PAO1K, CN573, PAV237, PAO1, A11, Is573 and Is580 with the different virulence factors, including three resistotypes of PAO1, PA7 and PA14 and on *K. pneumoniae* strains with five different capsular serotypes and one with KPC-3.
- The natural groups (cocktails) of phages largely performed well, inhibiting PRM growth either in synergy or in proto-cooperation. Each cocktail showed a killing effect against at least two non-propagating strains.
  — *P. aeruginosa* phages largely suppress in vitro PRM (phage-resistant mutant) growth, either synergistically or in proto-cooperation.
  — *K. pneumoniae* phages overcome the inhibitory effect of single phages, resulting in synergy or proto-cooperation.

This study adds to the existing knowledge of phage behavior in cocktails and the formulation of therapeutic phage preparations. In order to evaluate the therapeutic potential of phages and properly address the current need, experimental evidence and scientific practices need to be used, translated in vivo and communicated.

## 4. Materials and Methods

### 4.1. Bacterial growth

#### 4.1.1. Bacterial Growth Media

For *P. aeruginosa* and *K. pneumoniae* bacterial and phage culture, Lysogeny Broth (LB) (Lennox, L3022-1KG; Sigma-Aldrich, Burlington, MA, USA) and Tryptic Soy Broth (TSB) No. 2 (Millipore, Molsheim, France, 51228-500G-F) were used, respectively, with or without the addition of bacteriological agar (VWR Chemicals, Suwanee, GA, USA, # 9002-18-0).

#### 4.1.2. Bacterial Growth Methods and Conditions

Bacterial stocks were maintained in TSB broth with 15% glycerol at −80 °C. Bacterial cultures were routinely prepared on LB or TSB agar and incubated overnight at 32 °C. Bacterial suspensions were prepared in DPBS (Lonza™ BE17-512F, Walkersville, MD, USA) and diluted to an optical density (OD600, PerkinElmer Lambda 12 UV/VIS spectrometer, PerkinElmer, Macquarie Park, Australia) corresponding to the desired colony forming units cfu/mL (colony forming unit). For high-throughput screening, bacterial strains were cultured in 96-microtiter plates with LB or TSB, and OD was measured using a Spectra Thermo microplate reader (Tecan A-5082, Tecan, Grödig, Austria).

#### 4.1.3. Biological Materials

In total, 102 *P. aeruginosa* and 155 *K. pneumoniae* strains were used in this work. *P. aeruginosa* strains originated from the bacterial strain collection of the Laboratory for Molecular and Cellular Technology (LabMCT) of the Queen Astrid Military Hospital (Brussels, Belgium); *K. pneumoniae* strains from LabMCT, the laboratory of Gene Technology of KU Leuven (Heverlee, Belgium); the Fundamental and Applied Research for Animals & Health unit (FARAH, Liège, Belgium); the Faculty of Veterinary Medicine of Liege (Liège, Belgium); the Universitätsklinikum Jena (Jena, Germany); and the University of Jyväskylä (Jyväskylä, Finland). A detailed list of bacterial strains is provided in the Supplementary Materials (Tables S3 and S4). *P. aeruginosa* strains PAO1K (Ref.: ATCC 15692) [22], CN573 (Eliava IBMV collection), and PAV237 [25] and *K. pneumoniae* strains LabMCT0682, ATCC 27736, NCTC 13438, and SB4385 (Liege) were used as phage propagation hosts. *P. aeruginosa* strains PAO1 (Ref.: #16444); PA14 (Ref.: #8365); PA7 (Ref.: #6393), Is573; and A11, Is580 (all from LabMCT) and *K. pneumoniae* strains 10394 (Jena, Germany), 70165 (Jyväskylä, Finland) and VPKP389 (Jyväskylä, Finland) were used for phage lytic activity evaluation tests.

The phages used for *P. aeruginosa* were Atpa001, Atpa002, Atpa003, Atpa004, Atpa005, Atpa006, Atpa008, Atpa009, Atpa010, Atpa011, Atpa012, Atpa013, Atpa014. For *K. pneumoniae*, phages Atkp001, Atkp004, Atkp006, Atkp007, Atkp008, Atkp009, Atkp010, Atkp012, Atkp014, Atkp015, Atkp016 were used. All these phages were isolated for this study. *P. aeruginosa* phages Qatpa008, Qatpa009 and Qatpa010 were also included, isolated by the military hospital as part of the Inteliphages (BioWin) project.

### 4.2. Bacterial Enumeration (cfu) and Adjustment to OD

Bacterial enumeration was performed according to the standard procedure [26] with the following modifications: 10-fold se./rial dilutions of the bacterial suspension were made in a 96-microtiter plate with DPBS (180 µL + of 20 µL) diluted to 10^−7^. The OD was first measured using a microplate reader. These dilutions (20 µL) were pipetted with a multichannel pipette (4 drops in a row) on the solid agar surface across the column of the plate grid (3 + 3 columns on a square Petri dish). Three replicates were made for each test bacterium. Plates were incubated upside down at 32 °C for 18 h. The average number of individual colonies for different dilutions carried out in triplicate was calculated to obtain cfu/mL.

### 4.3. Phage Isolation

The phage enrichment (PE) method was used for new phage isolations, as previously described [26].

### 4.4. Phage Detection—Preliminary Test

The direct spot test on bacterial streaks (spot on streak) and the direct spot test on bacterial spots (spot on spot) were used to detect new phages in the lysates, as was previously described [18].

### 4.5. Phage Activity Detection—Phage Plaque Formation and Enumeration

The newly isolated phages were studied using the “Multiple Dilutions of phage(s) on a Single Plate (MD/SP)” technique, as previously described [27]. Briefly, ten-fold serial dilutions of phage lysates were made in 96-well microplates up to a dilution factor of 10^−8^. Three hundred microliters of bacterial suspensions with an OD600 of 0.3 (*P. aeruginosa*) or 0.2 (*K. pneumoniae*), corresponding to 10^8^ cfu/mL, were added to 8 mL melted (46 °C) soft agar (0.7%) in a 15 mL tube, and the mixture was spread on solid agar (1.5%) pre-prepared on square Petri dishes (Greiner, Noida, India, #688161). After drying (in a laminar flow), 2 µL of each phage dilution was spotted onto a soft agar surface across the column of the plate grid (six columns on a square Petri dish) using a multichannel pipette. Three replicates of each test phage were made. After all spots were completely absorbed into the top layer of agar (in a Biosafety Cabinet (BSC)), the test plates were incubated upside down at 32 °C for 18 h. The average number of plaques (plaque forming units, pfu) for the different dilutions and replicates was calculated and multiplied by 500 to obtain the number of plaques in 1 mL. This number was then multiplied by the reciprocal of the dilution to obtain the phage titer in pfu/mL.

### 4.6. Confirmatory Test for Phage Plaque Formation and Enumeration

For phage titer (cfu/mL) determination, the single dilutions on multiple plates (SD/MP) method was used [27]. Briefly, 10- or 100-fold serial dilutions of phage lysates were made in 96-well microplates up to a 10–10 dilution factor. One hundred microliters of bacterial suspensions with an OD600 of 0.3 (*P. aeruginosa*) or 0.2 (*K. pneumoniae*), corresponding to 10^+08^ cfu/mL, were added to a 5 mL tube containing 100 µL of a phage dilution. After 5 min (adsorption time), 3 mL of melted (46 °C) soft agar (0.7%) was added to the phage–bacteria mixture, and the tube contents were spread onto a solid agar (1.5%) plate (Greiner, #633181). One plate was prepared per phage dilution. After the top agar layer had solidified, the test plates were incubated upside down at 32 °C for 18 h. The average number of plaques for the different dilutions and replicates was calculated and multiplied by 10 to obtain the number of plaques in 1 mL. This number was then multiplied by the reciprocal of the dilution to obtain the phage titer in pfu/mL.

### 4.7. Phage Host Range and Efficiency of Plating (EOP)

EOP was studied using the MD/SP DAL method in triplicate. The average titer (pfu/mL) of a phage given on the test bacterial strain was compared to the average titer given on the propagating host bacterial strain. The average of EOP for each phage on a panel of different bacterial strains was then calculated.

### 4.8. Phage Plaque Purification

To differentiate individual plaques, a combination of three methods was used at different stages of purification. First, 1–2 rounds of the MD/SP method were used to pick up individual phage plaques. Then, the T-streaking technique [27] was used for individual plaque passaging (3 rounds). Briefly, 150 µL of bacterial suspension (10^+08^ cfu/mL) was mixed with 3 mL of melted (46 °C) soft agar (0.7%) and spread on a solid agar (1.5%) plate. After drying in a BSC for 15 min, the phage lysate was spotted in one corner of an upper agar layer, and the phage inoculum was then streaked sequentially across the upper agar surface in three segments using a sterile cotton swab. This reduced the number of phages in each segment, resulting in the formation of individual phage plaques that were separated and distanced from each other. At the final stage of plaque purification, the SD/MP method (5 rounds) was used as a confirmatory/validation test.

### 4.9. Preparation of High Concentrated Phage Using Web Pattern Plates [27,28]

First, the phage/bacteria ratio that produces a “web-pattern agar top” was determined for each phage using the MD/SP method. Typically, plates with low phage dilutions display a “clear pattern agar top”, indicating that all inoculated bacteria have been readily lysed and that, as a result, the maximum phage amplification capacity was not used up. In contrast, plates with high phage dilutions typically display a “spotted pattern agar top” with countable phage plaques, indicating that the phage amplification rate could not keep up with the bacterial growth rate. In between the last “clear pattern agar top” plate and the “spotted pattern agar top” plate is typically the optimal phage dilution (and phage/bacteria ratio), producing a “web pattern agar plate”, indicating that the phages have reached their maximum propagation efficiency.

After identifying the web pattern plate for each phage, a number of copies were made (depending on need and the plate size used (94 × 16 mm or 120 × 120 mm). The freshly made solid agar plates (solidified but not dried out, which is essential) were used to keep the plate contents moist during incubation and also easy to scrape off. First, the “web pattern mixture” was prepared based on the previously determined phage–bacteria ratio: 100 µL or 250 µL (depending on the plate size) of a phage suspension (corresponding to the “web pattern dilution”) was added to 100 µL or 250 µL of 10^+08^ cfu/mL bacteria and up to 3 mL or 8 mL of overlay agar (0.7%). The mixture was then shaken manually and spread over a solid agar surface. Once the top layer of agar had solidified, the plates were incubated at 32 °C for 18 h. The plates were not inverted to allow water to condense on the top layer of agar. After incubation, the top layer of agar was scraped from each plate separately using disposable L-shaped scrapers (VWR, 612-1561) and collected in 50 mL centrifuge tubes (Greiner 227261-N). These tubes were then centrifuged at 6000× *g* for 20 min and then filtered through 0.22 µm membrane filters (Millex^®^, SLGPR33RB, Sigma-Aldrich, Burlington, MA, USA). The resulting phage lysates were partially purified by ultracentrifugation (T-647.5 Fixed Angle Rotor, Thermo Scientific, Norristown, PA, USA) at 25,500 rpm (K factor 418). After centrifugation, the supernatants were discarded, and the pellets were resuspended in 3 mL DPBS (Lonza™ BE17-512F) and stored at 4 °C until further use.

### 4.10. Phage DNA Isolation

The Invitrogen™ PureLink™ Viral RNA/DNA Mini Kit (REF: 12280-050, Fisher Scientific, Waltham, MA, USA) was used for phage DNA isolation, according to the supplier’s protocol. Samples were first pre-treated with DNase I and RNase to remove the residual DNA and RNA from the bacterial host cells [29]. Specifically, 450 µL of phage lysate was mixed with 50 µL DNase I 10 × buffer, 8 µL DNase I (1 U/µL, Thermo Scientific™, EN0525) and 4 µL RNase A (10 mg/mL, Thermo Scientific™, EN0531) and incubated at 37° C for 40 min. Then, 20 µL of 50 mM EDTA (Thermo Scientific™, EN0525) was added to neutralize DNase I and RNase A.

### 4.11. Phage Genome Sequencing

Phage genome sequencing was performed on an Illumina MiniSeq machine (Illumina, San Diego, CA, USA) using a paired-end approach (2 × 150 bp), as described in Eskenazi et al., 2022. Quality of the sequencing and trimming prior to assembly were performed as described in [30]. For assembly, the genomes were constructed using Unicycler (v0.4.8). Assemblies were inspected using Bandage (v0.8.1).

### 4.12. Phage Liquid Culturing (PLC)

#### 4.12.1. Appelmans’ Method

**Figure 17:**
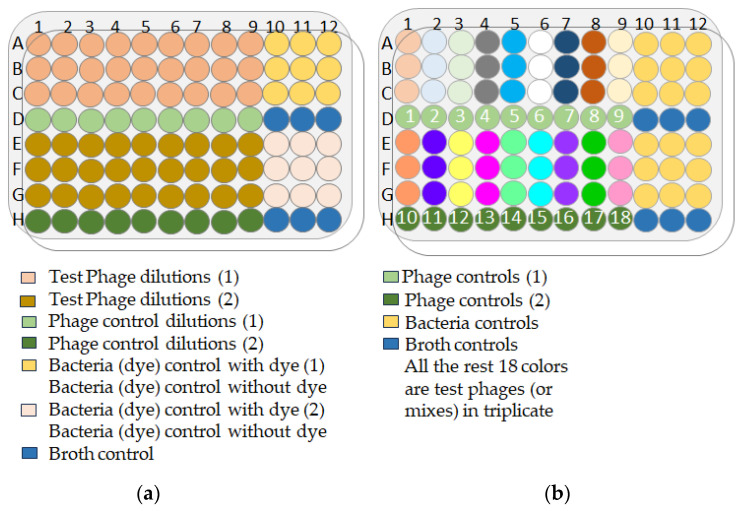
(**a**) OmniLog plate layout for the Appelmans’ method; (**b**) OmniLog plate layout for the lytic activity evaluation of individual phages or phage mixtures.

Ninety-six-well microtiter plates were prepared as follows: 180 µL of LB was added to each test well. Ten-fold serial dilutions of phage samples were made to a dilution factor of 10-9. Twenty microliters of bacterial suspension with an OD600 of 0.003 (*P. aeruginosa*) or 0.002 (*K. pneumoniae*), corresponding to 2.10 + 6 cfu/mL, was added to each test well (except the “phage only” and “LB only” control wells). Finally, 100% *v*/*v* Redox Dye Mix H (100X) (Biolog #74228) was added to all wells at a final concentration of 1%. The plate layout (Figure 17a) included “phage only”, “bacteria only” and “LB only” controls, and all experiments and controls were performed in triplicate wells. Test plates were immediately incubated in the OmniLog system (Biolog, Hayward, CA, USA) at 37 °C for 24 h or 48 h. A possible reduction (causing a color change) in the tetrazolium dye due to bacterial respiration was monitored and recorded every 15 min by the OmniLog system. Bacterial growth is expressed in relative respiration units (rru).

#### 4.12.2. Phage (or Mixture) Lytic Activity Evaluation Using Phage Liquid Culturing (PLC)

The optimal phage–bacteria ratio was determined according to the Appelmans protocol. A 96-well microtiter plate was used for one bacterial strain at a particular dilution. The plates were prepared as follows (Figure 17b): 180 µL of bacterial suspension in LB was added to each well (except the “phage only” and “LB only” wells). Then, 20 µL of phage (or phage mixture) suspension at a given dilution was added to each test and “phage only” control well. The remainder of the protocol was identical to that described in the Appelmans method protocol (Section 4.12.1).

#### 4.12.3. Enumeration of Bacteria and Phage upon Completion of the OmniLog Experiments (Figure 18)

Enumeration of bacteria (cfu/mL) and phage (pfu/mL) was performed after 48 h of incubation (endpoint) in the OmniLog system. Initially, 20 µL were aspirated from each test well (triplicate) using a multichannel pipette and transferred to a 96-well microtiter plate with DPBS (180 µL) to obtain a 10-fold dilution. Further dilutions and counts (cfu/mL) were performed as described above (Section 2.2). Second, the remaining contents of the wells in the OmniLog plates were filtered using 96-well filter plates (MultiScreen-GV Filter Plate, Millipore, Molsheim, France, 0.22 µm, clear, sterile, #MSGVS2210), which were centrifuged (Eppendorf centrifuge 5810, A-4-62 swing bucket rotor, Eppendorf, Hamburg, Germany) at 4000 rpm for two minutes. The supernatants were collected in a new 96-microtiter plate, and the contents of each well were subjected to the MD/SP method to determine phage activity (pfu/mL).

### 4.13. Statistical Analyses

Statistical analyses were performed using Prism 10.0.0. (GraphPad Software). The results are shown as mean ± SEM, and *p* < 0.05 was considered statistically significant.

## Figures and Tables

**Figure 3:**
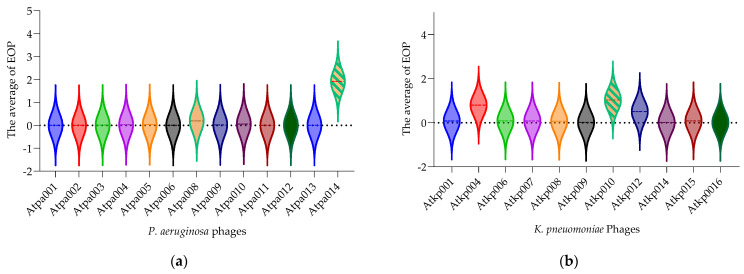
(**a**) EOP of *P. aeruginosa* phages; (**b**) EOP of *K. pneumonia* phages. Each violon plot shows the average of each phage EOP on the tested bacterial panel.

**Figure 18:**
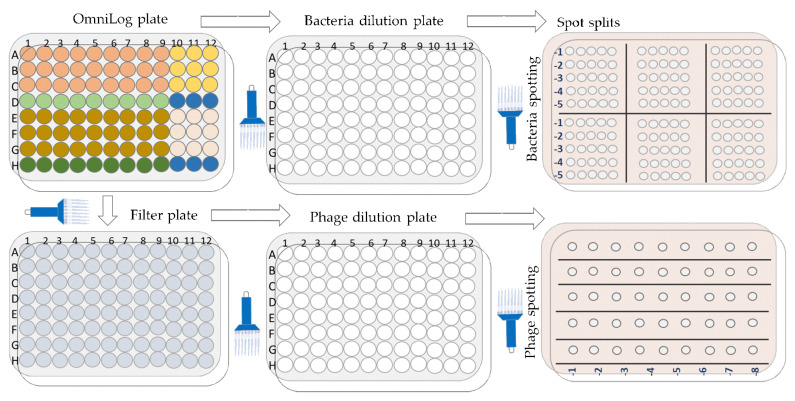
Schematic overview of the bacterial and phage enumeration work flow.

**Table 1:** Isolation host and sources, plaque morphology and genome characteristics of *P. aeruginosa* phages.

Phage Name	Isolation Host Strains (*P. aeruginosa*)	Isolation Source	Plaque Morphology	Genome Size (bp)	Genus
Atpa001	PAO1K ATCC 15692 DSM 22644	Brussels International Airport sewage water	2 mm Ø (diameter) plaque	66,468	*Hollowayvirus*
Atpa002	PAO1K ATCC 15692 DSM 22644	Brussels International Airport sewage water	2 mm Ø plaque	65,922	*Pbunavirus*
Atpa003	PAO1K ATCC 15692 DSM 22644	Brussels International Airport sewage water	1 mm Ø clear plaque with halo	60,912	*Yuavirus*
Atpa004	PAO1K ATCC 15692 DSM 22644	Aquafin, Brussels, Belgium	4 mm Ø plaque with size polymorphism, clear center and irregular margin of 2.5 mm	72,290	*Litunavirus*
Atpa005	PAO1K ATCC 15692 DSM 22644	Academic Hospital Monika, Brussels, Belgium	4 mm Ø plaque with size polymorphism, clear center and irregular margin of 2.5 mm	72,348	*Litunavirus*
Atpa006	PAO1K ATCC 15692 DSM 22644	Academic Hospital Monika, Brussels, Belgium	1 mm Ø translucent plaque with irregular margin	45,608	*Bruynoghevirus*
Atpa008	PAO1K ATCC 15692 DSM 22644	Academic Hospital Monika, Brussels, Belgium	2 mm Ø shiny turbid plaque with irregular margin	93,506	*Samunavirus*
Atpa009	CN573 DSM 22644 Eliava IBMV Collection CN573	Saint-Jean hospital, Brussels, Belgium	3 mm Ø plaque with clear center, size polymorphism and an irregular margin of 2 mm width	71,982	*Litunavirus*
Atpa010	PAO1K ATCC 15692 DSM 22644	River water from Nepal	6–7 mm Ø clear plaque with clear center and an irregular margin of 1 mm width	43,381	*Phikmvvirus*
Atpa011	PAO1K ATCC 15692 DSM 22644	River water from Nepal	6 mm Ø clear plaque with clear center and an irregular margin	Contig 1, 283,284 Contig 2, 43,363	*Phikzvirus Phikmvvirus*
Atpa012	PAO1K ATCC 15692 DSM 22644	River water from Congo	1 mm Ø plaque with size polymorphism	61,551	*Yuavirus*
Atpa013	Eliava IBMV Collection CN573	River water from Congo	0.85 mm Ø plaque	61,750	*Yuavirus*
Atpa014	CN573 DSM 22644 Eliava IBMV Collection CN573	River water from Congo	4 mm Ø plaque with 0.85 mm Ø clear center	45,479	*Bruynoghevirus*
Qatpa008	PAV237 Olympia, WA, USA	QAMH sewage water	2 mm Ø translucent plaque with size polymorphism and an irregular margin	63,083	New genus (unclassified)
Qatpa009	PAV237 Olympia, WA, USA	QAMH sewage water	Clear 1 mm Ø plaque with size polymorphism and an irregular margin	58,469	*Maxdohrnvirus*
Qatpa0010	CN573 DSM 22644 Eliava IBMV Collection CN573	QAMH sewage water	1 mm Ø clear plaque	Contig 11, cov 6.4, 87,990	*Pakpunavirus*

**Table 2:** Isolation hosts and sources, plaque morphology and genomic characteristics of *Klebsiella pneumoniae* phages.

Phage Name	Isolation Host Strains (*K. pneumoniae*)	Isolation Source	Plaque Morphology	Genome Size (bp)	Genus
Atkp01	LabMCT0682	Brussels International Airport sewage water	4 mm Ø plaque with size polymorphism, clear center and a margin of 3 mm width	52,977	New genus (*Drexlerviridae*)
Atkp04	LabMCT0682	Aquafin, Brussels, Belgium	3 mm Ø plaque with clear center and an irregular margin of 2 mm width	52,586	New genus (*Drexlerviridae*)
Atkp06	LabMCT0682	Aquafin, Brussels, Belgium	1 mm Ø translucent plaque	110,813	*Sugarlandvirus*
Atkp07	LabMCT0682	Academic Hospital Monika, Brussels, Belgium	1.5 mm Ø plaque with clear center	115,044	*Sugarlandvirus*
Atkp08	LabMCT0682	Academic Hospital Monika, Brussels, Belgium	1.5 mm Ø plaque with clear center and a margin of 0.5 mm width	115,044	*Sugarlandvirus*
Atkp09	NCTC 13438	Saint-Jean hospital, Brussels, Belgium	1.5 mm Ø plaque with clear center and a margin of 0.5 mm width	106,874	*Sugarlandvirus*
Atkp10	SB4385	Saint-Jean hospital, Brussels, Belgium	1 mm Ø plaque with clear center	108,212	*Sugarlandvirus*
Atkp12	ATCC 27736	River water from Nepal	3 mm Ø plaque with clear center and an irregular margin of 2 mm width	49,740	*Webervirus*
Atkp14	LabMCT0682	River water from Congo	Ø1 mm clear plaque	175,285	*Marfavirus*
Atkp15	LabMCT0682	River water from Congo	Ø1 mm clear plaque	176,764	*Pseudotevenvirus*
Atkp16	ATCC 27736	River water from Congo	Ø1 mm with twisted undulate margin	176,276	*Slopekvirus*

**Table 3:** Composition of *P. aeruginosa* phage cocktails.

Cocktail Number	1	2	3	4	5	6	7
Phage components and their genera	Atpa001 *Hollowayvirus*	Atpa001	Atpa005 *Litunavirus*	Atpa008 *Samunavirus*	Atpa010 *Phikmvvirus*	Atpa012 *Yuavirus*	Qatpa008 New genus (unclassified)
	Atpa002 *Pbunavirus*	Atpa003	Atpa006 *Bruynoghevirus*	Atpa009 *Litunavirus*	Atpa011 *Phikzvirus*	Atpa013 *Yuavirus*	Qatpa009 *Maxdohrnvirus*
	Atpa003 *Yuavirus*	Atpa004 *Litunavirus*	n/a	n/a	n/a	Atpa014 *Bruynoghevirus*	Qatpa010 *Pakpunavirus*
Isolation sources	Brussels International Airport sewage water (BIA)	BIA and Aquafin (A), Brussels, Belgium	Academic Hospital Monika, Brussels, Belgium	Saint-Jean hospital, Brussels, Belgium	River water from Nepal	River water from Congo	Queen Astrid Military Hospital sewage water

Description: n/a, not applicable.

**Table 4:** Composition of *K. pneumoniae* phage cocktails.

Cocktail Number	1	2	3	4	5	6	7
Phage component and their genera	Atkp001 New genus (*Drexlerviridae*)	Atkp001	Atkp004 New genus (*Drexlerviridae*)	Atkp007 *Sugarlandvirus*	Atkp009	Atkp012 *Webervirus*	Atkp014 *Marfavirus*
	Atkp008 *Sugarlandvirus*	Atkp009 *Sugarlandvirus*	Atkp006 *Sugarlandvirus*	Atkp008	Atkp010 *Sugarlandvirus*	Atkp016 *Slopekvirus*	Atkp015 *Pseudotevenvirus*
	n/a	Atpa004	n/a	n/a	n/a	n/a	Atkp016
Isolation source	BIA and Academic hospital Monika, Brussels, Belgium	BIA and Saint-Jean hospital, Brussels, Belgium	Aquafin, Brussels, Belgium	Academic hospital Monika, Brussels, Belgium	Saint-Jean hospital, Brussels, Belgium	River waters from Nepal and Congo	River waters from Congo

Description: n/a, not applicable.

**Table 5:** Interpretation of individual *P. aeruginosa* phage components and cocktail activity.

*P. aeruginosa* Strain rru Characteristics/ Parameters	Cocktail 1 Phage Components	Cocktail 2 Phage Components	Cocktail 3 Phage Components	Cocktail 4 Phage Components	Cocktail 5 Phage Components	Cocktail 6 Phage Components	Cocktail 7 Phage Components
PAO1K Control—221 rru	Cl-32 h, 161 rru --	Cl-42 h, 135 rru --	Cl-48 h, --, --	In-48 h, 135 rru --	Cl-48 h, --, --	Cl-48 h, --, --	Cl-30 h, 235 rru PRM
O5-serotype T3SS ExoS, ExoY oprI A1 oprL B11	Atpa001, Cl-12 h PRM, 319 rru (EOP 0.002)	Atpa001, Cl-12 h PRM, 319 rru (EOP 0.002)	Atpa005, Cl-28 h PRM, 283 rru (EOP 0.03)	Atpa008, Cl-10 h --, 208 rru (EOP 0.2)	Atpa010, Cl-48 h --, --, (EOP 0.06)	Atpa012, Cl-8 h PRM, 302 rru (EOP 0.007)	Qatpa008, Cl-30 h PRM, 245 rru (EOP 7.8)
	Atpa002, Cl-24 h PRM, 265 rru (EOP 0.0005)	Atpa003, Cl-12 h PRM, 248 rru (EOP 0.01)	Atpa006, Cl-28 h --, 237 rru (EOP 0.002)	Atpa009, Cl-24 h PRM, 307 rru (EOP 0.22)	Atpa011, Cl-48 h --, --, (EOP 0.002)	Atpa013, Cl-12 h PRM, 285 rru (EOP 0.003)	Qatpa009, Cl-12 h PRM, 301 rru (EOP 0.32)
	Atpa003, Cl-12 h PRM, 248 rru (EOP 0.01)	Atpa004, Cl-28 h PRM, 341 rru (EOP 0.03)	n/a	n/a	n/a	Atpa014, Cl-16 h --, 235 rru (EOP 1.9)	Qatpa010, Cl-16 h PRM, 307 rru (EOP 0.022)
Interpretation	Synergy	Synergy	Synergy	Antagonism	Proto- cooperation	Synergy	Proto- cooperation
PAO1 Control—208 rru	Cl-48 h, --, --	n/a	Cl-48 h, --, --	n/a	n/a	n/a	n/a
O5-serotype T3SS ExoS, ExoY oprI A1 oprL B11	Atpa001, Cl-6 h --, 231 rru (EOP 0.002)	n/a	Atpa005, Cl-15 h --, 199 rru (EOP 0.03)	n/a	n/a	n/a	n/a
	Atpa002, Cl-12 h --, 188 rru (EOP 0.0005)	n/a	Atpa006, Cl-8 h PRM, 188 rru (EOP 0.002)	n/a	n/a	n/a	n/a
	Atpa003, Cl-8 h PRM, 294 rru (EOP 0.01)	n/a	n/a	n/a	n/a	n/a	n/a
Interpretation	Synergy	-	Synergy	n/a	n/a	n/a	n/a
CN573 Control—222 rru	Cl-48 h, --, --	Cl-48 h, --, --	Cl-48 h, --, --	Cl-48 h, --, --	Cl-48 h, --, --	Cl-48 h, --, --	Cl-20 h, 187 rru --
O1-serotype oprI B1 oprL B09 ExoS	Atpa001, Cl-28 h --, 234 rru (EOP 0.002)	Atpa001, Cl-28 h --, 234 rru (EOP 0.002)	Atpa005m Cl-48 h --, --, (EOP 0.03)	Atpa008, Cl-18 h --, 189 rru (EOP 0.2)	Atpa010, Cl-48 h --, --, (EOP 0.06)	Atpa012, ln-36 h --, 204 rru (EOP 0.007)	Qatpa008, Cl-28 h --, 122 rru (EOP 7.8)
	Atpa002, Cl-36 h --, 225 rru (EOP 0.0005)	Atpa003, Cl-28 h PRM, 265 rru (EOP 0.01)	Atpa006, Cl-48 h --, --, (EOP 0.002)	Atpa009, Cl-48 h --, --, (EOP 0.22)	Atpa011, Cl-48 h --, --, (EOP 0.002)	Atpa013, Cl-30 h PRM, 248 rru (EOP 0.003)	Qatpa009, Cl-12 h PRM, 252 rru (EOP 0.32)
	Atpa003, Cl-28 h PRM, 265 rru (EOP 0.01)	Atpa004, Cl-48 h --, --, (EOP 0.03)	n/a	n/a	n/a	Atpa014, Cl-14 h --, 265 rru (EOP 1.9)	Qatpa010, Cl-48 h --, --, (EOP 0.022)
Interpretation	Synergy	Proto-cooperation	Proto- cooperation	Proto- cooperation	Proto- cooperation	Synergy	Antagonism
PAV237 Control—228 rru	Cl-20 h, 255 rru PRM	Cl-48 h, --, --	Cl-48 h, --, --	Cl-48 h, --, --	Cl-48 h, --, --	Cl-48 h, --, --	Cl-8 h, 296 rru PRM
*lasB* gene coding for elastase B	Atpa001, Cl-20 h --, 224 rru (EOP 0.002)	Atpa001, Cl-20 h --, 224 rru (EOP 0.002)	Atpa005, Cl-48 h --, (EOP 0.03)	Atpa008, Cl-12 h --, 213 rru (EOP 0.2)	Atpa010, Cl-14 h --, 209 rru (EOP 0.06)	Atpa012, Cl-6 h --, 220 rru (EOP 0.007)	Qatpa008, Cl-20 h PRM, 271 rru (EOP 7.8)
	Atpa002, Cl-10 h PRM, 263 rru (EOP 0.0005)	Atpa003, Cl-10 h PRM, 285 rru (EOP 0.01)	Atpa006, Cl-12 h --, 193 rru (EOP 0.002)	Atpa009, Cl-48 h --, --, (EOP 0.22)	Atpa011, Cl-48 h --, --, (EOP 0.002)	Atpa013, Cl-10 h --, 179 rru (EOP 0.003)	Qatpa009, Cl-8 h PRM, 295 rru (EOP0.32)
	Atpa003, Cl-10 h PRM, 285 rru (EOP 0.01)	Atpa004, Cl-48 h --, --, (EOP 0.03)	n/a	n/a	n/a	Atpa014, Cl-48 h --, --, (EOP 1.9)	Qatpa010, Cl-14 h --, 209 rru (EOP 0.022)
Interpretation	Proto- cooperation	Proto- cooperation	Proto-cooperation	Proto- cooperation	Proto- cooperation	Proto- cooperation	Antagonism
PA14 Control—185 rru	n/a	n/a	Cl-48 h, --, --	n/a	Cl-48 h, --, --	n/a	n/a
O10-serotype, oprI B1 oprL A05 T3SS ExoU, ExoY	n/a	n/a	Atpa005, Cl-6 h --, 180 rru (EOP 0.03)	n/a	Atpa010, Cl-12 h --, 193 rru (EOP 0.06)	n/a	n/a
	-	-	Atpa006, Cl-24 h PRM, 245 rru (EOP 0.002)	-	Atpa011, Cl-26 h PRM, 193 rru (EOP 0.002)	-	-
Interpretation	-	-	Synergy	-	Synergy	-	-
Is573 Control—248 rru	n/a	n/a	n/a	n/a	n/a	Cl-48 h, --, --	Cl-30 h, 205 rru PRM
O11-serotype, oprI B1 oprL B02 ExoU	n/a	n/a	n/a	n/a	n/a	Atpa012, Cl-6 h --, 239 rru (EOP 0.007)	Qatpa008, Cl-16 h --, 225 rru (EOP 7.8)
	n/a	n/a	n/a	n/a	n/a	Atpa013, Cl-8 h --, 257 rru (EOP 0.003)	Qatpa009, Cl-14 h --, 242 rru (EOP 0.32)
	n/a	n/a	n/a	n/a	n/a	Atpa014, Cl-48 h --, --, (EOP 1.9)	Qatpa010, Cl-8 h --, 249 rru (EOP 0.022)
Interpretation	-	-	-	-	-	Proto- cooperation	Synergy
A11 Control—241 rru	Cl-28 h, 216 rru --	n/a	n/a	n/a	Cl-30 h, 210 rru --	n/a	n/a
oprI B1 oprL B12 ExoS	Atpa001, --, --, 250 rru (EOP 0.002)	n/a	n/a	n/a	Atpa010, Cl-6 h --, 250 rru (EOP 0.06)	n/a	n/a
	Atpa002, Cl-6 h --, 198 rru (EOP 0.0005)	n/a	n/a	n/a	Atpa011, Cl-26 h --, 252 rru (EOP 0.002)	n/a	n/a
	Atpa003, Cl-8 h --, 197 rru (EOP 0.01)	n/a	n/a	n/a	n/a	n/a	n/a
Interpretation	Synergy	n/a	n/a	n/a	Synergy	n/a	n/a
Is580 Control—222 rru	n/a	n/a	n/a	Cl-44 h, 148 rru	Cl-48 h	n/a	n/a
O3-serotype oprI B1 oprL B03 ExoS	n/a	n/a	n/a	Atpa008, Cl-6 h --, 188 rru (EOP 0.2)	Atpa010, Cl-10 h --, 213 rru (EOP 0.06)	n/a	n/a
	n/a	n/a	n/a	Atpa009, Cl-10 h --, 193 rru (EOP 0.22)	Atpa011, Cl-48 h --, --, (EOP 0.002)	n/a	n/a
Interpretation	n/a	n/a	n/a	Synergy	Proto- cooperation	n/a	n/a
PA7 Control—261 rru	Cl-10 h, 246 rru --	--, 298 rru PRM	--, 212 rru --	--, 283 rru PRM	Cl-14 h, 296 rru PRM	Cl-8 h, 247 rru --	Cl-10 h, 297 rru PRM
MDR O12-serotype TPS ExlA oprI F1 oprL E03	Atpa001, --, PRM, 280 rru (EOP 0.002)	Atpa001, --, --, 280 rru (EOP 0.002)	Atpa005 --, 256 rru (EOP 0.03)	Atpa008, --, --, 236 rru (EOP 0.2)	Atpa010, --, PRM, 275 rru (EOP 0.06)	Atpa012, --, PRM, 287 rru (EOP 0.007)	Qatpa008, Cl-20 h PRM, 275 rru (EOP 7.8)
	Atpa002, --, --, 255 rru (EOP 0.0005)	Atpa003, --, PRM, 284 rru (EOP 0.01)	Atpa006, --, --, 208 rru (EOP 0.002)	Atpa009, --, --, 227 rru (EOP 0.22)	Atpa011, Cl-14 h PRM, 305 rru (EOP 0.002)	Atpa013, --, PRM, 288 rru (EOP 0.003)	Qatpa009, --, --, 236 rru (EOP 0.32)
	Atpa003, --, --, 284 rru (EOP 0.01)	Atpa004, --, --, 265 rru (EOP 0.03)	n/a	n/a	n/a	Atpa014, Cl-8 h --, 274 rru (EOP 1.9)	Qatpa010, Cl-10 h --, 297 rru (EOP 0.022)
Interpretation	Synergy	Antagonism	-	Antagonism	Proto-cooperation	Proto- cooperation	Antagonism

Description: Each first raw gives a cocktail activity, followed by the individual phage components; in each cell, the activity is described as follows: CL or In or --. PRM or --, rru or --, (EOP value). Abbreviations: CL, clearing; In, inhibition; rru, relative respiration unit; PRM, phage-resistant mutant; --, not available; n/a, not applicable.

**Table 6:** Interpretation of individual *K. pneumoniae* phage components and cocktail activity.

*K. pneumoniae* Strains/ Capsule Type	Cocktail 1 Phage Components	Cocktail 2 Phage Components	Cocktail 3 Phage Components	Cocktail 4 Phage Components	Cocktail 5 Phage Components	Cocktail 6 Phage Components	Cocktail 7 Phage Components
LabMCT 0682 Control—192 rru	Cl-48 h, --, --	Cl-48 h, --, --	Cl-48 h, --, --	Cl-48 h, --, --	Cl-48 h, --, --	Cl-48 h, --, --	Cl-48 h, --, --
Capsular serotype 81	Atkp001, In-48 h --, 163 rru (EOP 0.08)	Atkp001, In-48 h --, 163 rru (EOP 0.08)	Atkp004, Cl-48 h --, --, (EOP 0.8)	Atkp007, ln-48 h --, 136 rru (EOP 0.08)	Atkp00,9 CL-48 h --, --, (EOP 0.013)	Atkp012, Cl-48 h --, --, (EOP 0.5)	Atkp014, Cl-48 h --, --, EOP 0.011)
	Atkp008, In-48 h --, 16 rru (EOP 0.07)	Atkp009, In-48 h --, 106 rru (EOP 0.013)	Atkp006, In-48 h --, 180 rru (EOP 0.09)	Atkp008, In-48 h --, 116 rru (EOP 0.07)	Atkp010, Cl-48 h --, --, (EOP 1.02)	Atkp016, Cl-48 h --, --, (EOP 0.015)	Atkp015, In-48 h --, 112 rru (EOP 0.09)
	n/a	n/a	n/a	n/a	n/a	n/a	Atkp016, Cl-48 h --, --, (EOP 0.015)
Interpretation	Synergy	Synergy	Proto-cooperation	Synergy	Proto-cooperation	Proto-cooperation	Proto-cooperation
Atcc 27736 Control—250 rru	n/a	Cl-8 h, 178 rru --,	n/a	n/a	n/a	Cl-48 h, --, --	Cl-48 h, --, --
Capsular serotype 30	n/a	Atkp001, --, --, 250 rru (EOP 0.08)	n/a	n/a	n/a	Atkp012, Cl-48 h --, --, (EOP 0.5)	Atkp014, Cl-12 h --, 218 rru (EOP 0.011)
	n/a	Atkp009, --, --, 250 rru (EOP 0.013)	n/a	n/a	n/a	Atkp016, Cl-48 h --, --, EOP 0.015	Atkp015, --, --, --, (EOP 0.09)
	n/a	n/a	n/a	n/a	n/a	n/a	Atkp016, Cl-48 h --, --, (EOP 0.015)
Interpretation	n/a	Synergy	n/a	n/a	n/a	Proto-cooperation	Proto-cooperation
nctc 13438 Control—272 rru	n/a	n/a	n/a	n/a	n/a	Cl-48 h, --, --, --,	Cl-48 h, --, --, --,
carbapenemase KPC-3	n/a	n/a	n/a	n/a	n/a	Atkp012, --, --, --, (EOP 0.5)	Atkp014, --, --, --, (EOP 0.011)
	n/a	n/a	n/a	n/a	n/a	Atkp016, Cl-16 h --, 201 rru (EOP 0.015)	Atkp015, --, --, --, (EOP 0.09)
	n/a	n/a	n/a	n/a	n/a	n/a	Atkp016, Cl-16 h --, 201 rru (EOP 0.015)
interpretation	n/a	n/a	n/a	n/a	n/a	Synergy	Synergy
SB4385 Control—255 rru	n/a	n/a	n/a	n/a	n/a	Cl-48 h, --, --,	Cl-48 h, --, --,
Capsular serotype 1	n/a	n/a	n/a	n/a	n/a	Atkp012, Cl-6 h --, --, (EOP 0.5)	Atkp014, --, --, --, (EOP 0.011)
	n/a	n/a	n/a	n/a	n/a	Atkp016, Cl-48 h --, --, (EOP 0.015)	Atkp015, --, --, --, (EOP 0.09)
	n/a	n/a	n/a	n/a	n/a	n/a	Atkp016 Cl-48 h EOP 0.015
interpretation	n/a	n/a	n/a	n/a	n/a	Proto-cooperation	Proto-cooperation
10394 Control—265 rru	n/a	n/a	n/a	n/a	n/a	In-48 h, 206 rru --	In-48 h, 185 rru --
Capsular serotype 62	n/a	n/a	n/a	n/a	n/a	Atkp012, --, PRM, 341 rru (EOP 0.5)	Atkp014, --, --, 258 rru (EOP 0.011)
	n/a	n/a	n/a	n/a	n/a	Atkp016, --, --, 266 rru (EOP 0.015)	Atkp015, --, --, 272 rru (EOP 0.09)
	n/a	n/a	n/a	n/a	n/a	n/a	Atkp016, --, --, 266 rru (EOP 0.015)
	n/a	n/a	n/a	n/a	n/a	Synergy	Synergy
70165 Control—065 rru	n/a	ln-48 h, 194 rru --	ln-48 h, 202 rru --	Cl-10 h, ln-48 h, --, 216 rru	In-48 h, 185 rru --,	n/a	n/a
Capsular serotype 2	n/a	Atkp001, --, --, 224 rru (EOP 0.08)	Atkp004, ln-48 h --, 201 rru (EOP0.8)	Atkp007, Cl-8 h --, 257 rru (EOP 0.08)	Atkp010, In-48 h --, 185 rru (EOP 1.02)	n/a	n/a
	n/a	Atkp009, --, --, 246 rru (EOP 0.013)	Atkp006, --, --, 246 rru (EOP 0.08)	Atkp008, Cl-8 h --, 243 rru (EOP 0.07)	Atkp009, --, --, 246 rru (EOP 0.013)	n/a	n/a
interpretation	n/a	Synergy	Proto-cooperation	Synergy	Proto-cooperation	n/a	n/a
VPKP389 Control—294 rru	Cl-48 h, --, --	Cl-28 h, --, --	n/a	n/a	Cl-48 h, --, --	n/a	n/a
Capsular serotype 27	Atkp001, --, ---, --, (EOP 0.08)	Atkp001, --, --, --, (EOP0.08)	n/a	n/a	Atkp009, Cl-48 h --, --, (EOP0.013)	n/a	n/a
	Atkp008 Cl-12 h EOP 0.08	Atkp009 In-48 h EOP 0.013	n/a	n/a	Atkp010, --, --, 275 rru (EOP 0.5)	n/a	n/a
Interpretation	Synergy	Synergy	n/a	n/a	Proto-cooperation	n/a	n/a

Description: Each first raw gives a cocktail activity, followed by the individual phage components; in each cell, the activity is described as follows: CL or In or --. PRM or --, rru or --, (EOP value). Abbreviations: CL, clearing; In, inhibition; rru, relative respiration unit; PRM, phage-resistant mutant; --, not available; n/a, not applicable.

## Data Availability

Data are contained within the article and Supplementary Materials.

## Supplementary Materials

The following supporting information can be downloaded at: https://www.mdpi.com/article/10.3390/antibiotics13050385/s1.

## References

[1] Haines M.E.K., Hodges F.E., Nale J.Y., Mahony J., van Sinderen D., Kaczorowska J., Alrashid B., Akter M., Brown N., Sauvageau D. (2021). Analysis of Selection Methods to Develop Novel Phage Therapy Cocktails Against Antimicrobial Resistant Clinical Isolates of Bacteria. Front. Microbiol..

[2] WHO Regional Office for Europe/European Centre for Disease Prevention and Control (2022). Antimicrobial Resistance Surveillance in Europe 2022–2020 Data. Copenhagen: WHO Regional Office for Europe. https://www.ecdc.europa.eu/en/publications-data/antimicrobial-resistance-surveillance-europe-2022-2020-data.

[3] European Centre for Disease Prevention and Control (2019). Surveillance of antimicrobial resistance in Europe 2018. Stockholm: ECDC. https://www.ecdc.europa.eu/sites/default/files/documents/surveillance-antimicrobial-resistance-Europe-2018.pdf.

[4] Nikolich M.P., Filippov A.A. (2020). Bacteriophage Therapy: Developments and Directions. Antibiotics.

[5] Pirnay J.P., Djebara S., Steurs G., Griselain J., Cochez C., De Soir S., Glonti T., Spiessens A., Vanden Berghe E., Green S. (2023). Retrospective, observational analysis of the first one hundred consecutive cases of personalized bacteriophage therapy of difficult-to-treat infections facilitated by a Belgian consortium. medRxiv.

[6] Chan B.K., Turner P.E., Kim S., Mojibian H.R., Elefteriades J.A., Narayan D. (2018). Phage treatment of an aortic graft infected with Pseudomonas aeruginosa. Evolut. Med. Public Health.

[7] Forti F., Roach D.R., Cafora M., Pasini M.E., Horner D.S., Fiscarelli E.V., Rossitto M., Cariani L., Briani F., Debarbieux L. (2018). Design of a Broad-Range Bacteriophage Cocktail That Reduces Pseudomonas aeruginosa Biofilms and Treats Acute Infections in Two Animal Models. Antimicrob. Agents Chemother..

[8] Martins W.M.B.S., Li M., Sands K., Lenzi M.H., Portal E., Mathias J., Dantas P.P., Migliavacca R., Hunter J.R., Medeiros E.A. (2022). Effective phage cocktail to combat the rising incidence of extensively drug-resistant *Klebsiella pneumoniae* sequence type 16. Emerg. Microbes Infect..

[9] Liang B., Han B., Shi Y., Li X., Zhao W., Kastelic J., Gao J. (2023). Effective of phage cocktail against Klebsiella pneumoniae infection of murine mammary glands. Microb. Pathog..

[10] Ribeiro J.M., Ribeiro J.M., Pereira G.N., Pereira G.N., Junior I.D., Junior I.D., Teixeira G.M., Teixeira G.M., Bertozzi M.M., Bertozzi M.M. (2023). Comparative analysis of effectiveness for phage cocktail development against multiple Salmonella serovars and its biofilm control activity. Sci. Rep..

[11] Guerrero-Bustamante C.A., Dedrick R.M., Garlena R.A., Russell D.A., Hatfull G.F. (2021). Toward a Phage Cocktail for Tuberculosis: Susceptibility and Tuberculocidal Action of Mycobacteriophages against Diverse Mycobacterium tuberculosis Strains. mBio.

[12] Naknaen A., Samernate T., Wannasrichan W., Surachat K., Nonejuie P., Chaikeeratisak V. (2023). Combination of genetically diverse Pseudomonas phages enhances the cocktail efficiency against bacteria. Sci. Rep..

[13] Abedon S.T., Danis-Wlodarczyk K.M., Wozniak D.J. (2021). Phage Cocktail Development for Bacteriophage Therapy: Toward Improving Spectrum of Activity Breadth and Depth. Pharmaceuticals.

[14] Markwitz P., Lood C., Olszak T., van Noort V., Lavigne R., Drulis-Kawa Z. (2022). Genome-driven elucidation of phage-host interplay and impact of phage resistance evolution on bacterial fitness. ISME J..

[15] Behzadi P., Baráth Z., Gajdács M. (2021). It’s Not Easy Being Green: A Narrative Review on the Microbiology, Virulence and Therapeutic Prospects of Multidrug-Resistant *Pseudomonas aeruginosa*. Antibiotics.

[16] Reboud E., Basso P., Maillard A.P., Huber P., Attrée I. (2017). Exolysin Shapes the Virulence of *Pseudomonas aeruginosa* Clonal Outliers. Toxins.

[17] Pirnay J.-P., Bilocq F., Pot B., Cornelis P., Zizi M., Van Eldere J., Deschaght P., Vaneechoutte M., Jennes S., Pitt T. (2009). Pseudomonas aeruginosa Population Structure Revisited. PLoS ONE.

[18] Grace A., Sahu R., Owen D.R., Dennis V.A. (2022). Pseudomonas aeruginosa reference strains PAO1 and PA14: A genomic, phenotypic, and therapeutic review. Front. Microbiol..

[19] Imbert P.R., Louche A., Luizet J., Grandjean T., Bigot S., Wood T.E., Gagné S., Blanco A., Wunderley L., Terradot L. (2017). A *Pseudomonas aeruginosa* TIR effector mediates immune evasion by targeting UBAP1 and TLR adaptors. EMBO J..

[20] Khan M., Stapleton F., Summers S., Rice S.A., Willcox M.D.P. (2020). Antibiotic Resistance Characteristics of *Pseudomonas aeruginosa* Isolated from Keratitis in Australia and India. Antibiotics.

[21] Subedi D., Vijay A.K., Kohli G.S., Rice S.A., Willcox M. (2018). Association between possession of ExoU and antibiotic resistance in Pseudomonas aeruginosa. PLoS ONE.

[22] Ammann C.G., Nagl M., Nogler M., Coraça-Huber D.C. (2016). Pseudomonas aeruginosa outcompetes other bacteria in the manifestation and maintenance of a biofilm in polyvinylchloride tubing as used in dental devices. Arch. Microbiol..

[23] Stover C.K., Pham X.Q., Erwin A.L., Mizoguchi S.D., Warrener P., Hickey M.J., Brinkman F.S.L., Hufnagle W.O., Kowalik D.J., Lagrou M. (2000). Complete genome sequence of Pseudomonas aeruginosa PAO1, an opportunistic pathogen. Nature.

[24] Chibeu A., Ceyssens P.-J., Hertveldt K., Volckaert G., Cornelis P., Matthijs S., Lavigne R. (2009). The adsorption of Pseudomonas aeruginosa bacteriophage phiKMV is dependent on expression regulation of type IV pili genes. FEMS Microbiol. Lett..

[25] Antoine C., Laforêt F., Blasdel B., Glonti T., Kutter E., Pirnay J., Mainil J., Delcenserie V., Thiry D. (2021). Efficacy assessment of PEV2 phage on Galleria mellonella larvae infected with a Pseudomonas aeruginosa dog otitis isolate. Res. Veter Sci..

[26] Hazan R., Que Y.-A., Maura D., Rahme L.G. (2012). A method for high throughput determination of viable bacteria cell counts in 96-well plates. BMC Microbiol..

[27] Glonti T., Pirnay J.-P. (2022). In Vitro Techniques and Measurements of Phage Characteristics That Are Important for Phage Therapy Success. Viruses.

[28] Adams M.D. (1959). Bacteriophages.

[29] Jakočiūnė D., Moodley A. (2018). A Rapid Bacteriophage DNA Extraction Method. Methods Protoc..

[30] Eskenazi A., Lood C., Wubbolts J., Hites M., Balarjishvili N., Leshkasheli L., Askilashvili L., Kvachadze L., van Noort V., Wagemans J. (2022). Combination of pre-adapted bacteriophage therapy and antibiotics for treatment of fracture-related infection due to pandrug-resistant *Klebsiella pneumoniae*. Nat. Commun..

