# Supplementary material for: Use of the Naturally Occurring Bacteriophage Grouping Model for the Design of Potent Therapeutic Cocktails
Source: Antibiotics (Basel). 2024 Apr 24;13(5):385. doi: 10.3390/antibiotics13050385 (PMC11117255; doi:10.3390/antibiotics13050385)
Supplement: Supplementary file 1 [file antibiotics-13-00385-s001.zip › antibiotics-2965492-supplementary.pdf]

Table S1. P. aeruginosa strains list.

Original Name	Location	Country	Year (2)	Source	fAFLP	Serotype	*oprD*	*oprL*	*oprI*	*exoS/U*	*TfpO*	*fpvA*	*fpvB*	Genes mediating ABR	ABR group
PER08	Ghent	Belgium	1992	Wound	32	11	C202	B12	B1	*exoS*	NA	I	POS	PER-1/2, ANT(2'')	3
Bu002	Budapest	Hungary	1997	Wound	24	11	B107	B12	B1	*exoS*	$TfpO_b$	II b	POS	ANT(3'')-Ia	4
9AR3	De Haan	Belgium	1993	CF-patient		NT	B110	B07	B1	*exoS*	$TfpO_b$	III	POS	NA	2
PHLS08959 (Liverpool)	Liverpool	UK	2003	CF-patient	20	NT	B108	B02	B1	*exoS*	$TfpO_b$	III	POS	NA	3
MC161	Leuven	Belgium	2003	CF-patient	20	NT	B101	B12	B1	*exoS*	$TfpO_b$	III	POS	NA	TRM
PHLS08916 (Midlands I)	Birmingham	UK	2003	CF-patient	20	NT	B107	B12	B1	*exoS*	$TfpO_b$	III	POS	NA	4
13BR3	De Haan	Belgium	1993	CF-patient		NT	B115	B02	A1	*exoS*	NA	III	POS	NA	2
Li010	Lisbon	Portugal	1997	CF-patient		NT	B104 (DOM6)	A07	B1	*exoS*	$TfpO_b$	III	POS	NA	5
So095	Sofia	Bulgaria	1997	Burn		NT	C106	B12	B1	*exoS*	NA	III	POS	NA	0
Bo546	Boston	USA	1992	Burn		12	B114	B15	E1	*exoS*	NA	I	POS	GES-1/2, TEM-like, AAC(3)-IIa	3
O12-20	Roehampton	UK	1987	Clinical non CF		12	C103	B11	A1	*exoS*	$TfpO_b$	I	POS	ANT(3'')-Ia	1
So098	Sofia	Bulgaria	1997	Wound	37	12	A111	B08	B1	*exoS*	$TfpO_b$	III	POS	PSE, AAC(6')-Ib	
O12-17	Pordenone	Italy	1988	Clinical non CF	37	12	A111	B08	B1	*exoS*	$TfpO_b$	III	POS	PSE, AAC(6')-Ib	
Co7388	Cali	Colombia	1999	Urine	37	12	A111	B08	B1	*exoS*	$TfpO_b$	III	POS	VIM-2-like	
Co380791	Cali	Colombia	2003	Blood	37	12	A111	B08	B1	*exoS*	$TfpO_b$	III	POS	VIM-2-like, AAC(6')-Ib	
1709-12	Leuven	Belgium	2004	Clinical non CF	37	12	A111 (DOM20)	B08	B1	*exoS*	$TfpO_b$	III	POS	PSE, AAC(6')-Ib	5
1709-20	Leuven	Belgium	2004	Clinical non CF	37	12	A111 (DOM20)	B08	B1	*exoS*	$TfpO_b$	III	POS	PSE, AAC(6')-Ib	5
MC110	Brussels	Belgium	2003	CF-patient	20	NT	B101	B02	A1	*exoS*	$TfpO_b$	I	POS	NA	2
CPHL 10701	Surrey	UK	1967	Sputum	20	NT	B107	B07	A1	*exoS*	$TfpO_b$	I	POS	NA	1
MC361 (blue)	Brussels	Belgium	2003	CF-patient	20	NT	B112	B05	B1	*exoS*	$TfpO_b$	I	POS	NA	2
MC116	Brussels	Belgium	2003	CF-patient	31	NT	B107 (DOM17)	B12	B1	*exoS*	$TfpO_b$	I	POS	NA	2
MC305	Ghent	Belgium	2003	CF-patient	31	NT	B101	B02	A1	*exoS*	$TfpO_b$	I	POS	NA	1

Clone M	Hanover	Germany	1994	CF-patient	20	NT	B106 (DOM15)	B12	B1	*exoS*	*TfpO*$_b$	I	POS	NA	1
LiA11/2004	Almada	Portugal	2004	Cat nose	36	9	B101	B02	A1	*exoS*	*TfpO*$_b$	I	POS	NA	2
CPHL 2000	London	UK	1923	Wound	30	9	B113	B05	A1	*exoS*	*TfpO*$_b$	I	POS	NA	0
CPHL 1999	London	UK	1924	Ear	30	NT	C103	B11	A1	*exoS*	NA	I	POS	NA	0
TA21	Hobart	Australia	2004	Sputum	20	NT	C108	B02	A1	*exoS*	NA	I	POS	NA	2
PN1352(65)w	Panama City	Panama	2006	Nose	20	NT	C103	B11	A1	*exoS*	NA	I	POS	NA	1
NCF013	Hobart	Australia	2003	Lung carcinoma	20	NT	C103	B11	A1	*exoS*	NA	I	POS	NA	1
NCF015	Hobart	Australia	2003	COPD	20	NT	C106	B12	A1	*exoS*	NA	I	POS	NA	1
TA28	Hobart	Australia	2004	Wound	20	NT	C109	B12	B1	*exoS*	NA	I	POS	NA	2
MC178 (LCV)	Brussels	Belgium	2003	CF-patient	34	NT	B107	B11	A1	*exoS*	NA	I	POS	NA	1
MC178 (SCV)	Brussels	Belgium	2003	CF-patient	34	NT	B107	B11	A1	*exoS*	NA	I	POS	NA	TRM
C2 (clone C)	Hannover	Germany	1988	CF-patient	28	1/13	B106	B02	B1	*exoS*	NA	II b	POS	NA	2
PA7	Buenos Aires	Argentina	pre 1984	Wound		12	A204	E03	F1	NA	NA	II b	NA	NA	4
SIS3740 (O12)	Athens	Greece	1994	Sputum	38	12	A111	B08	B1	*exoS*	*TfpO*$_b$	III	POS	PSE, AAC(6')-Ib	4
PER05	Ghent	Belgium	1999	Sputum	38	12	A111	B08	B1	*exoS*	*TfpO*$_b$	III	POS	PER-1/2	2
Br667 (AFLP 35)	Brussels	Belgium	1998	Burn	33	12	A111	B08	B1	*exoS*	*TfpO*$_b$	III	POS	PSE, AAC(6')-Ib	4
Br993	Brussels	Belgium	1999	Sputum	33	12	A111 (DOM13)	B08	B1	*exoS*	*TfpO*$_b$	III	POS	PSE, AAC(6')-Ib	5
Lo049	London	UK	1996	Burn	33	12	A111	B08	B1	*exoS*	*TfpO*$_b$	III	POS	PSE, AAC(6')-Ib	4
KAT3529 (O12)	Athens	Greece	1994	Wound	39	12	A111 (DOM16)	B08	B1	*exoS*	*TfpO*$_b$	III	POS	AAC(6')-Ib	4
Is586(13T)	Istanbul	Turkey	1997	Burn	39	12	A111	B08	B1	*exoS*	*TfpO*$_b$	III	POS	PSE, AAC(6')-Ib	4
A10	Paris	France	1882-1918	Wound	28	1	B103	B12	B1	*exoS*	*TfpO*$_b$	I	POS	NA	0
10BR1	De Haan	Belgium	1993	CF-patient	14	1	B107	B12	B1	*exoS*	NA	I	POS	NA	2
Aa249	Aachen	Germany	1997	Burn	30	NT	B107 (DOM7)	B12	B1	*exoS*	*TfpO*$_b$	I	POS	AAC(6')-Ib	5
Bu004	Budapest	Hungary	1997	Throat	32	NT	C106	B12	A1	*exoS*	NA	I	POS	NA	1
Bo548	Boston	USA	1992	Burn		NT	C107 (DOM12)	B12	A1	*exoS*	NA	I	POS	NA	2
So099	Sofia	Bulgaria	1997	Burn		NT	C104	B01	A1	*exoS*	NA	I	POS	AAC(6')-Ib	4

NCF017	Hobart	Australia	2003	Sputum	43	NT	C103	B12	B1	*exoS*	$TfpO_b$	I	POS	NA	1
Is579	Istanbul	Turkey	1997	Burn	30	8	B112	B02	B1	*exoS*	NA	IIa**	POS	NA	3
PHLS08960 (Manchester)	Manchester	UK	2003	CF-patient	7	NT	B107 (DOM14)	B02	A1	*exoS*	$TfpO_b$	II b	POS	NA	4
TA03	Hobart	Australia	2004	Wound	20	NT	A113	B02	B1	*exoS*	NA	II b	POS	NA	1
A20	Paris	France	1882-1918	Wound	28	NT	B101	B12	B1	*exoS*	$TfpO_b$	II b	POS	NA	0
Lw1047	Lwiro	Congo	2001	Blood	30	NT	B107	B02	A1	*exoS*	$TfpO_b$	II b	POS	ANT(3")-Ia	4
MC084	Antwerp	Belgium	2003	CF-patient	20	NT	B106	B02	B1	*exoS*	NA	II b	POS	NA	1
MC039	Ghent	Belgium	2003	CF-patient	20	NT	B107 (DOM18)	B12	B1	*exoS*	NA	II b	POS	NA	5
MC093	Leuven	Belgium	2003	CF-patient	21	NT	B106	B02	B1	*exoS*	NA	II b	POS	NA	2
MC325	Ghent	Belgium	2003	CF-patient	21	NT	B106 (DOM19)	B02	B1	*exoS*	NA	II b	POS	NA	4
MC075	Antwerp	Belgium	2003	CF-patient	21	NT	B106	B02	B1	*exoS*	$TfpO_b$	II b	POS	NA	1
MC142	Brussels	Belgium	2003	CF-patient	20	NT	B110	B01	B1	*exoS*	$TfpO_b$	II b	POS	NA	3
MC099	Ghent	Belgium	2003	CF-patient	20	NT	B101	B02	B1	*exoS*	$TfpO_b$	II b	POS	NA	3
MC299	Brussels	Belgium	2003	CF-patient	21	1	B106	B02	B1	*exoS*	NA	II b	POS	NA	1
C13 (clone C)	Hannover	Germany	1985	CF-patient	28	1	B106	B02	B1	*exoS*	NA	II b	POS	NA	0
U018A (CF type 4)	Hobart	Australia	2003	CF-patient	20	1	B110	B12	B1	*exoS*	$TfpO_b$	II b	POS	NA	1
C1 (clone C)	Hannover	Germany	1987	CF-patient	28	1/13	B106	B02	B1	*exoS*	NA	II b	POS	NA	2
U003A (CF type 4)	Hobart	Australia	2003	CF-patient	43	1	B110	B12	B1	*exoS*	$TfpO_b$	II b	POS	NA	4
TA08	Hobart	Australia	2003	Sputum	20	3	B107	B12	B1	*exoS*	NA	II b	POS	NA	1
So103	Sofia	Bulgaria	1997	Wound	9	11	C202	B02	B1	*exoU*	NA	II b	POS	OXA-group I, AAC(6')-Ib	3
Aa245	Aachen	Germany	1997	Burn	11	11	C202 (DOM3)	B02	B1	*exoU*	NA	II b	POS	GES-1/2, TEM-like, AAC(6')-Ib	5
TA34	Hobart	Australia	2004	Urine	22	NT	B107	B07	A1	*exoS*	NA	II b	POS	NA	2
A11	Paris	France	1882-1918	Wound	28	NT	B101	B12	B1	*exoS*	$TfpO_b$	II b	POS	NA	0
A16	Paris	France	1882-1918	Wound	28	NT	B101	B12	B1	*exoS*	$TfpO_b$	II b	POS	NA	0
A13	Paris	France	1882-1918	Wound	28	NT	B101	B12	B1	*exoS*	$TfpO_b$	II b	POS	NA	0
PhDW6	Tacloban City	The Philippines	1993	Wound		NT	B101	B13	A1	*exoS*	$TfpO_b$	II b	POS	NA	2

A22	Paris	France	1882-1918	Wound	20	6	B107	B12	B1	*exoS*	$TfpO_b$	II b	POS	NA	1
Br670	Brussels	Belgium	1998	Sputum	30	6	B107 (DOM8)	B07	B1	*exoS*	$TfpO_b$	II b	POS	SHV	3
Br776	Brussels	Belgium	1998	Throat		6	B110	B12	B1	*exoS*	$TfpO_b$	II b	POS	NA	4
CPHL 6749	Elstree	UK	1944	Urine	20	6	C103	B12	B1	*exoS*	$TfpO_b$	I	POS	NA	1
AES1	Australia	Australia	1999	CF-patient		6	B119	B02	A1	*exoS*	$TfpO_b$	III	POS	NA	1
M-184	Bucarest	Romania	1965-1978	Faeces	7	1/3/10/13	B107	B02	A1	*exoS*	NA	II a	POS	NA	1
Is580	Istanbul	Turkey	1997	Burn	30	3	B108	B03	B1	*exoS*	NA	II a	POS	NA	4
M-280	Bucarest	Romania	1965-1978	Faeces	7	3	B107	B02	A1	*exoS*	NA	II a	POS	NA	1
M-79	Bucarest	Romania	1965-1978	Urine	7	NT	B107	B02	A1	*exoS*	$TfpO_b$	II a	POS	NA	1
5BR2	De Haan	Belgium	1993	CF-patient	20	NT	C109	B07	B1	*exoS*	NA	II a	POS	NA	2
C5311 (RAPD A097)	Vancouver	Canada	2002	CF-patient	20	NT	B107	B13	B1	*exoS*	$TfpO_b$	II a	POS	NA	3
TA04	Hobart	Australia	2003	Foot ulcer	20	NT	B107	B07	A1	*exoS*	$TfpO_b$	II a	POS	NA	1
MC096	Leuven	Belgium	2003	CF-patient	20	NT	B106	B12	B1	*exoS*	$TfpO_b$	II a	POS	NA	0
MC361 (green)	Brussels	Belgium	2003	CF-patient	20	NT	B107	B02	B1	*exoS*	NA	II a	POS	NA	1
C3128 (RAPD A002)	Vancouver	Canada	2002	CF-patient	21	NT	B106	B02	B1	*exoS*	NA	II a	POS	NA	2
Br735 (AFLP 8)	Brussels	Belgium	1998	Burn		NT	C204	A05	B1	*exoS*	NA	II a	POS	PSE	1
Br680	Brussels	Belgium	1998	Burn		12	A201	E01	F1	*exoS*	$TfpO_b$	II b	NA	NA	0
AGO4092 (O11)	Athens	Greece	1994	Urine	12	11	C202	B02	B1	*exoU*	NA	II b	POS	SHV, ANT(3")-Ia, AAC(6')-Ib	
PN1296(62)	Panama City	Panama	2006	Wound		11	C202 (DOM21)	B02	B1	*exoU*	NA	II a	POS	TEM-like	5
PAO1	Melbourne	Australia	1955	Wound	30	5	C103	B11	A1	*exoS*	NA	I	POS	NA	1
A18	Paris	France	1882-1918	Leg ulcer	20	6	B107	B12	C1	*exoS*	$TfpO_b$	I	POS	NA	1
MC086	Ghent	Belgium	2003	CF-patient	20	6	C106	B02	B1	*exoS*	$TfpO_b$	I	POS	NA	1
TA05	Hobart	Australia	2003	Sputum	26	6	B107	B12	B1	*exoS*	$TfpO_b$	I	POS	NA	1
Pr317	Prague	Czech Republic	1996	Burn		11	C202 (DOM10)	B02	B1	*exoU*	NA	II b	POS	ANT(3")-Ia, AAC(3)-IIa	
Br678	Brussels	Belgium	1998	Burn		11	C202 (DOM13)	B02	B1	*exoS+U*	NA	II b**	POS	PER-1/2	5

Us450	San Antonio	USA	1993	Burn	35	11	C103	B02	B1	*exoU*	*TfpO*b	II a	POS	AAC(6')-Ib	1
Us365	San Antonio	USA	1986	Wound	35	11	C202	B02	B1	*exoU*	NA	II a	POS	AAC(6')-Ib	0
Bo559	Boston	USA	1997	Burn		1	A103	B06	A1	*exoU*	*TfpO*b	I	POS	NA	1

Table S2. K. pneumoniae strains list.

#	LMG number	Original Name	Place of origin	Source of isolation	Capsule type	Virulence genes
1	A110050	1711-040762	Erazme Hospital, Brussels	Bone infection	K20	-
2	A110046	025187127-1	PTMC, CHU Nantes, France	Burn infection	N/A	-
3	17d0862(1)	-	UZGent	Burn wound	K24	-
4	A210120	36336	LHUB-ULB	Bone infection	N/A	-
5	A210157	ATCC 13883	UZGent	-	K3	-
6	ATCC 27736	ATCC 27736	LabMCT	-	N/A	-
7	SB4551	SB4551	LabMCT	-	N/A	-
8	S4 4385	S4 4385	LabMCT	-	N/A	-
9	130528/0682	-	LabMCT	Nez	N/A	-
10	121231/0018	-	LabMCT	-	K3	-
11	130306/0842	-	LabMCT	-	K81	-
12	110628/0752	-	LabMCT	-	K14	-
13	*121120/0608*	-	LabMCT	-	K28	-
14	080708/0488-2	-	LabMCT	-	N/A	-
15	*21209/0028*	-	LabMCT	-	N/A	-
16	110607/1254	-	LabMCT	-	N/A	-
17	A310226	2101260700 _original	LabMCT	Peritoneal fluid	N/A	-
18	A310229	121114/0502	VUB, Zizi	Peritoneal fluid	N/A	-
19	A310240	1D	HUDERF, Brussels	Peritoneal fluid	N/A	-
20	A310241	2D	HUDERF, Brussels	Peritoneal fluid	N/A	-
21	A310242	1C	HUDERF, Brussels	Peritoneal fluid	N/A	-
22	A310243	2C	HUDERF, Brussels	Peritoneal fluid	N/A	-
23	A310244	2103230964	HUDERF, Brussels	Peritoneal fluid	N/A	-
24	A310245	2103230968	HUDERF, Brussels	Peritoneal fluid	N/A	-
25	A310246	2103230970	HUDERF, Brussels	rectum	N/A	-
26	A310247	2103230966	HUDERF, Brussels	rectum	N/A	-
27	A310270	11819	Saint Luc	rectum	N/A	-
28	A310271	12063	Saint Luc	rectum	N/A	-
29	A310272	12146	Saint Luc	rectum	N/A	-
30	A310273	12307	Saint Luc	rectum	N/A	-
31	A310274	12351	Saint Luc	rectum	N/A	-

32	A310275	12820	Saint Luc	rectum	N/A	-
33	A310276	12894	Saint Luc	rectum	N/A	-
34	A310277	13010	Saint Luc	rectum	N/A	-
35	A310278	13021	Saint Luc	selles	N/A	-
36	A310279	13231	Saint Luc	rectum	N/A	-
37	A310280	13123	Saint Luc	selles	N/A	-
38	A310281	13134	Saint Luc	selles	N/A	-
39	A310282	13196	Saint Luc	rectum	N/A	-
40	A310283	13345	Saint Luc	rectum	N/A	-
41	A310284	13498	Saint Luc	rectum	N/A	-
42	A310285	13571	Saint Luc	rectum	N/A	-
43	A310286	13765	Saint Luc	rectum	N/A	-
44	A310287	14076	Saint Luc	rectum	N/A	-
45	A310288	14094	Saint Luc	rectum	N/A	-
46	A310289	14506	Saint Luc	rectum	N/A	-
47	A310290	14977	Saint Luc	rectum	N/A	-
48	A310291	14987	Saint Luc	rectum	N/A	-
49	A310292	15102	Saint Luc	rectum	N/A	-
50	A310293	CPE199	Saint Luc	rectum	N/A	-
51	A310294	CPE262	Saint Luc	rectum	N/A	-
52	A310295	CPE293	Saint Luc	carriage before surgery		-
53	A310296	CPE357	Saint Luc	-	N/A	-
54	A310360	OPTICS 1 Kp	UCL	-	N/A	-
55	10383	60fe7b3cd37098409a7e71e0	Universitats Klinikum Jena Germany	-	K62	-
56	10384	60fe7b3cd37098409a7e71e3	Universitats Klinikum Jena Germany	-	K62	-
57	10385	60fe7b3cd37098409a7e71e5	Universitats Klinikum Jena Germany	-	K4	-
58	10386	60fe7b3cd37098409a7e71e8	Universitats Klinikum Jena Germany	-	K2	-
59	10387	60fe7b3cd37098409a7e71ed	Universitats Klinikum Jena Germany	-	K62	-
60	10388	60fe7b3cd37098409a7e71ef	Universitats Klinikum Jena Germany	-	KL112	-
61	10389	60fe7b3cd37098409a7e71f0	Universitats Klinikum Jena Germany	-	K4	-
62	10390	60fe7b3cd37098409a7e71f1	Universitats Klinikum Jena Germany	-	K24	-
63	10391	60fe7b3cd37098409a7e71f6	Universitats Klinikum Jena Germany	-	K62	-

64	10392	60fe7b3cd37098409a7e71f8	Universitats Klinikum Jena Germany	-	K2	-
65	10393	60fe7b3cd37098409a7e71f9	Universitats Klinikum Jena Germany	-	K62	-
66	10394	60fe7b3cd37098409a7e71fa	Universitats Klinikum Jena Germany	-	K62	-
67	10395	60fe7b3cd37098409a7e71fc	Universitats Klinikum Jena Germany	-	K74	-
68	10396	60fe7b3cd37098409a7e7200	Universitats Klinikum Jena Germany	-	KL112	-
69	10397	60fe7b3cd37098409a7e7205	Universitats Klinikum Jena Germany	-	K2	-
70	10398	60fe7b3cd37098409a7e7206	Universitats Klinikum Jena Germany	-	KL112	-
71	10399	60fe7b3cd37098409a7e7207	Universitats Klinikum Jena Germany	-	KL112	-
72	10400	60fe7b3cd37098409a7e7208	Universitats Klinikum Jena Germany	-	K74	-
73	10401	60fe7b3cd37098409a7e7209	Universitats Klinikum Jena Germany	-	K2	-
74	10402	60fe7b3cd37098409a7e721d	Universitats Klinikum Jena Germany	-	KL107	-
75	10403	60fe7b3cd37098409a7e721e	Universitats Klinikum Jena Germany	-	K62	-
76	10404	60fe7b3cd37098409a7e7225	Universitats Klinikum Jena Germany	-	K68	-
77	10405	60fe7b3cd37098409a7e722b	Universitats Klinikum Jena Germany	-	K38	-
78	10406	60fe7b3cd37098409a7e722e	Universitats Klinikum Jena Germany	-	KL107	-
79	10407	60fe7b3cd37098409a7e723d	Universitats Klinikum Jena Germany	-	KL112	-
80	10408	60fe7b3cd37098409a7e71e2	Universitats Klinikum Jena Germany	-	KL120	-
81	10409	60fe7b3cd37098409a7e7245	Universitats Klinikum Jena Germany	-	KL102	-
82	10410	60fe82b3d37098409a7e726d	Universitats Klinikum Jena Germany	-	K24	-
83	10411	60fe82b3d37098409a7e725c	Universitats Klinikum Jena Germany	-	KL108	-
84	10412	60fe82b3d37098409a7e725b	Universitats Klinikum Jena Germany	-	K17	-
85	10413	60fe82b3d37098409a7e725a	Universitats Klinikum Jena Germany	-	KL105	-
86	10414	60fe82b3d37098409a7e7259	Universitats Klinikum Jena Germany	-	KL108	-

87	10415	60fe82b3d37098409a7e7258	Universitats Klinikum Jena Germany	-	K17	-
88	10416	60fe82b3d37098409a7e7257	Universitats Klinikum Jena Germany	-	KL105	-
89	10417	60fe82b3d37098409a7e725e	Universitats Klinikum Jena Germany	-	K24	-
90	10418	60fe82b3d37098409a7e7250	Universitats Klinikum Jena Germany	-	K17	-
91	10419	60fe82b3d37098409a7e724f	Universitats Klinikum Jena Germany	-	KL106	-
92	10420	60fe82b3d37098409a7e7256	Universitats Klinikum Jena Germany	-	KL105	-
93	10421	60fe82b3d37098409a7e7255	Universitats Klinikum Jena Germany	-	K64	-
94	10422	60fe82b3d37098409a7e7254	Universitats Klinikum Jena Germany	-	K2	-
95	10423	60fe82b3d37098409a7e7253	Universitats Klinikum Jena Germany	-	KL156-D1	-
96	10424	60fe82b3d37098409a7e726e	Universitats Klinikum Jena Germany	-	K30	-
97	10425	60fe82b3d37098409a7e726c	Universitats Klinikum Jena Germany	-	K45	-
98	10426	60fe82b3d37098409a7e726b	Universitats Klinikum Jena Germany	-	KL156-D1	-
99	10427	60fe82b3d37098409a7e726a	Universitats Klinikum Jena Germany	-	K55	-
100	10428	60fe7b3cd37098409a7e724c	Universitats Klinikum Jena Germany	-	K5	-
101	10429	60fe7b3cd37098409a7e724d	Universitats Klinikum Jena Germany	-	K57	-
102	10430	60fe7b3cd37098409a7e724e	Universitats Klinikum Jena Germany	-	K38	-
103	10431	61375150a181bb3680e24dc3	Universitats Klinikum Jena Germany	-	K16	-
104	10432	619e0fe79f46174c9eed71e1	Universitats Klinikum Jena Germany	-	K17	-
105	10433	619e0fe79f46174c9eed71e2	Universitats Klinikum Jena Germany	-	KL102	-
106	10434	619e0fe79f46174c9eed71e3	Universitats Klinikum Jena Germany	-	KL107	-
107	10435	619e0fe79f46174c9eed71e4	Universitats Klinikum Jena Germany	-	KL102	-
108	10436	619e0fe79f46174c9eed71e5	Universitats Klinikum Jena Germany	-	K24	-
109	10437	619e0fe79f46174c9eed71e6	Universitats Klinikum Jena Germany	-	K17	-

110	10438	619e0fe79f46174c9eed71e7	Universitats Klinikum Jena Germany	-	K17	-
111	10439	619e0fe79f46174c9eed71e8	Universitats Klinikum Jena Germany	-	KL141	-
112	10440	619e0fe79f46174c9eed71ea	Universitats Klinikum Jena Germany	-	K2	-
113	10441	619e0fe79f46174c9eed71eb	Universitats Klinikum Jena Germany	-	KL106	-
114	10442	619e0fe79f46174c9eed71ec	Universitats Klinikum Jena Germany	-	KL156-D1	-
115	106 KPK2	10924	University of Jyväskylä (FIN)	-	N/A	mrK
116	107 KPK3	70165	University of Jyväskylä (FIN)	-	K2	irp, fyu, ybt, kfu, mrk
117	108 KPK4	70415	University of Jyväskylä (FIN)	-	K2	mrk
118	109 KPK5	70708	University of Jyväskylä (FIN)	-	N/A (K15K17K50K51K52)	mrk
119	110 KPK6	71076	University of Jyväskylä (FIN)	-	K17	irp, mrk, fyu, ybt, kfu
120	111 KPK7	2008024	University of Jyväskylä (FIN)	-	K2	mrk, kfu
121	112 KPK8	2008025	University of Jyväskylä (FIN)	-	K13	mrk
122	113 KPK10	AO-8053	University of Jyväskylä (FIN)	-	N/A	mrk
123	114 KPK11	AO-15200	University of Jyväskylä (FIN)	-	K64,K14	mrk
124	115 KPK13	VPKP267	University of Jyväskylä (FIN)	-	K64,K15	mrk
125	116 KPK14	VPKP205	University of Jyväskylä (FIN)	-	K25	irp, mrk, ybt
126	117 KPK15	VPKP284	University of Jyväskylä (FIN)	-	K21	fyu, ybt, all, arc, glx
127	118 KPK16	VPKP374	University of Jyväskylä (FIN)	-	k2	irp, fyu, ybt, mrk
128	119 KPK17	VPKP430	University of Jyväskylä (FIN)	-	k3	irp, fyu, ybt, kfu, mrk
129	120 KPK18	VPKP389	University of Jyväskylä (FIN)	-	k27	irp, mrk, fyu, ybt
130	121 KPK20	VPKP229	University of Jyväskylä (FIN)	-	k25	irp, mrk, ybt
131	122 KPK24	ED502873	University of Jyväskylä (FIN)	-	N/A (K15K17K50K51K52)	irp, mrk, fyu, ybt
132	123 KPK25	N12	University of Jyväskylä (FIN)	-	K51	kfu, mrk
133	124 KPK3r2	-	University of Jyväskylä (FIN)	-	N/A	-
134	125 PT-KP1	-	University of Jyväskylä (FIN)	-	N/A	-
135	126 PT-KP2	-	University of Jyväskylä (FIN)	-	N/A	-
136	127 PT-KP3	-	University of Jyväskylä (FIN)	-	N/A	-
137	128 PT-KP4	-	University of Jyväskylä (FIN)	-	N/A	-
138	129 PT-KP5	-	University of Jyväskylä (FIN)	-	N/A	-
139	130 PT-KP8	-	University of Jyväskylä (FIN)	-	N/A	-

140	131 PT-KP11	-	University of Jyväskylä (FIN)	-	N/A	-
141	132 PT-KP12	-	University of Jyväskylä (FIN)	-	N/A	-
142	133 PT-KP13	-	University of Jyväskylä (FIN)	-	N/A	-
143	134 PT-KP14	-	University of Jyväskylä (FIN)	-	N/A	-
144	135 KP2r2	-	University of Jyväskylä (FIN)	-	N/A	-
145	136 KP2r3	-	University of Jyväskylä (FIN)	-	N/A	-
146	137 KP2r4	-	University of Jyväskylä (FIN)	-	N/A	-
147	138 NKP1	-	University of Jyväskylä (FIN)	-	N/A	-
148	139 NKP2	-	University of Jyväskylä (FIN)	-	N/A	-
149	140 NKP20	-	University of Jyväskylä (FIN)	-	N/A	-
150	141 EKP3	-	University of Jyväskylä (FIN)	-	N/A	-
151	142 EKP8	-	University of Jyväskylä (FIN)	-	N/A	-
152	143 EKP10	-	University of Jyväskylä (FIN)	-	N/A	-
153	144 EKP11	-	University of Jyväskylä (FIN)	-	N/A	-
154	145 PT-KO1	-	University of Jyväskylä (FIN)	-	N/A	-
155	146 KPK1	1534	University of Jyväskylä (FIN)	-	K15K17K50K51K52	-

Table S3. Characteristics and interpretation of OmniLog propagation curves for combinations of *P. aeruginosa* strains and individual phages and phage cocktails.

**P. aeruginosa Strain rru Characteristics/parameters**	**Cocktail 1** **Phage Components**	**Cocktail 2** **Phage components**	**Cocktail 3** **Phage components**	**Cocktail 4** **Phage components**	**Cocktail 5** **Phage components**	**Cocktail 6** **Phage components**	**Cocktail 7** **Phage components**
**PAO1K**<br>**221 rru**	Cl-32h<br>161 rru	Cl-42h<br>135 rru	Cl-48h	In-48h<br>135 rru	Cl-48h	Cl-48h	Cl-30h<br>PRM, 235 rru
**O5-serotype**<br>**T3SS**<br>**ExoS**<br>**oprI A1 oprL B11**	Atpa001<br>Cl-12h<br>PRM,<br>319 rru	Atpa001<br>Cl-12h<br>PRM,<br>319 rru	Atpa005<br>Cl-28h<br>PRMM,<br>283 rru	Atpa008<br>Cl-10h<br>208 rru	Atpa010<br>Cl-48h	Atpa012<br>Cl-8h<br>PRM,<br>302 rru	Qatpa008<br>Cl-30h<br>PRM,<br>245 rru
	Atpa002<br>Cl-24h<br>PRM, 265 rru	Atpa003<br>Cl-12h<br>PRM, 248 rru	Atpa006<br>Cl-28h<br>237 rru	Atpa009<br>Cl-24h<br>PRM, 307 rru	Atpa011<br>Cl-48h	Atpa013<br>Cl-12h<br>PRM, 285 rru	Qatpa009<br>Cl-12h<br>PRM, 301 rru
	Atpa003<br>Cl-12h<br>PRM, 248 rru	Atpa004<br>Cl-28h<br>PRM, 341 rru	n/a	n/a	n/a	Atpa014<br>Cl-16h<br>235 rru	Qatpa010<br>Cl-16h<br>PRM, 307 rru

Interpratation	Synergy: Increased clearing effect timing.	Synergy: Increased clearing effect timing.	Synergy: Increased clearing effect timing.	Antagonism or not sufficient pfu/ml: Shorten clearing effect timing.	Proto-cooperation: Clearing effect timing is the same.	Synergy: Increased clearing effect timing.	Proto-cooperation: Clearing effect timing is the same.
**PAO1 M, 208 rru**	Cl-48h	ndh	Cl-48h	ndh	ndh	ndh	ndh
**O5-serotype T3SS ExoS oprI A1 oprL B11**	Atpa001 Cl-6h 231 rru	ndh	Atpa005 Cl-15h 199 rru	ndh	ndh	ndh	ndh
	Atpa002 Cl-12h 188 rru	ndh	Atpa006 Cl-8h PRM, 188 rru	ndh	ndh	ndh	ndh
	Atpa003 Cl-8h PRM, 294 rru	ndh	n/a	ndh	ndh	ndh	ndh
**Interpratation**	Synergy: Increased clearing effect timing.	-	Synergy: Increased clearing effect timing.	ndh	ndh	ndh	ndh
**CN573 222 rru**	Cl-48h	Cl-48h	Cl-48h	Cl-48h	Cl-48h	Cl-48h	Cl-20h 187 rru
**O1-serotype oprI B1 oprL B09 ExoS**	Atpa001 Cl-28h 234 rru	Atpa001 Cl-28h 234 rru	Atpa005 Cl-48h	Atpa008 Cl-18h 189 rru	Atpa010 Cl-48h	Atpa012 ln-36h 204 rru	Qatpa008 Cl-28h 122 rru
	Atpa002 Cl-36h 225 rru	Atpa003 Cl-28h PRM, 265 rru	Atpa006 Cl-48h	Atpa009 Cl-48h	Atpa011 Cl-48h	Atpa013 Cl-30h PRM, 248 rru	Qatpa009 Cl-12h PRM, 252 rru
	Atpa003 Cl-28h PRM, 265 rru	Atpa004 Cl-48h	n/a	n/a	n/a	Atpa014 Cl-14h 265 rru	Qatpa010 Cl-48h
**Interpratation**	Synergy: Increased clearing effect timing.	Proto-cooperation: Clearing effect timing is the same.	Proto-cooperation: Clearing effect timing is the same.	Proto-cooperation: Clearing effect timing is the same.	Proto-cooperation: Clearing effect timing is the same.	Synergy: Increased clearing effect timing.	Antagonism or not sufficient pfu/ml: Shorten clearing effect timing
**PAV237 228 rru**	Cl-20h M, 255 rru	Cl-48h	Cl-48h	Cl-48h	Cl-48h	Cl-48h	Cl-8h PRM, 296 rru
	Atpa001 Cl-20h	Atpa001 Cl-20h	Atpa005 Cl-48h	Atpa008 Cl-12h	Atpa010 Cl-14h	Atpa012 Cl-6h	Qatpa008 Cl-20h PRM, 271 rru

	224 rru	224 rru		213 rru	209 rru	220 rru	
***lasB* gene coding for elastase B**	Atpa002<br>Cl-10h<br>PRM, 263 rru	Atpa003<br>Cl-10h<br>PRM, 285 rru	Atpa006<br>Cl-12h<br>193 rru	Atpa009<br>Cl-48h	Atpa011<br>Cl-48h	Atpa013<br>Cl-10h<br>179 rru	Qatpa009 Cl-8h<br>PRM, 295 rru
	Atpa003<br>Cl-10h<br>PRM, 285 rru	Atpa004<br>Cl-48h	n/a	n/a	n/a	Atpa014<br>Cl-48h	Qatpa010 Cl-14h<br>209 rru
**Interpratation**	<u>Proto-cooperation</u>: Clearing effect timing is the same.	<u>Proto-cooperation</u>: Clearing effect timing is the same.	<u>Proto-cooperation</u>: Clearing effect timing is the same.	<u>Proto-cooperation</u>: Clearing effect timing is the same.	<u>Proto-cooperation</u>: Clearing effect timing is the same.	<u>Proto-cooperation</u>: Clearing effect timing is the same.	<u>Antagonism or not sufficient pfu/ml</u>: Shorten clearing effect timing
**PA14<br>185 rru**	ndh	ndh	Cl-48h	ndh	Cl-48h	ndh	ndh
**O10-serotype, oprI B1 oprL A05 T3SS ExoU**	ndh	ndh	Atpa005<br>Cl-6h<br>180 rru	ndh	Atpa010<br>Cl-12h<br>193 rru	ndh	ndh
	-	-	Atpa006<br>Cl-24h<br>M, 245 rru	-	Atpa011<br>Cl-26h<br>M, 193 rru	-	-
**Interpratation**	-	-	<u>Synergy:</u><br>Increased clearing effect timing.	-	<u>Synergy:</u><br>Increased clearing effect timing.	-	-
**Is573<br>248 rru**	ndh	ndh	ndh	ndh	ndh	Cl-48h	Cl-30h<br>M, 205rru
**O11-serotype, oprI B1 oprL B02 ExoU**	ndh	ndh	ndh	ndh	ndh	Atpa012<br>Cl-6h<br>239 rru	Qatpa008<br>Cl-16h<br>225 rru
	ndh	ndh	ndh	ndh	ndh	Atpa013<br>Cl-8h<br>257 rru	Qatpa009 Cl-14h<br>242 rru
	ndh	ndh	ndh	ndh	ndh	Atpa014<br>Cl-48h	Qatpa010 Cl-8h<br>249 rru
**Interpratation**	-	-	-	-	-	<u>Protocooperation</u>: Clearing effect timing is the same.	<u>Synergy:</u><br>Increased clearing effect timing.
**A11<br>241 rru**	Cl-28h<br>216 rru	ndh	ndh	ndh	Cl-30h<br>210 rru	ndh	ndh
**oprI B1 oprL B12**	Atpa001<br>Cl-0h	ndh	ndh	ndh	Atpa010<br>Cl-6h	ndh	ndh

**ExoS**	250 rru				250 rru		
	Atpa002<br>Cl-6h<br>198 rru	ndh	ndh	ndh	Atpa011<br>Cl-26h<br>252 rru	ndh	ndh
	Atpa003<br>Cl-8h<br>197 rru	ndh	ndh	ndh	ndh	ndh	ndh
**Interpratation**	Synergy:<br>Increased clearing effect timing.	ndh	ndh	ndh	Synergy:<br>Increased clearing effect timing.	ndh	ndh
**Is580<br>222 rru**	ndh	ndh	ndh	Cl-44h<br>148 rru	Cl-48h	ndh	ndh
**O3-serotype<br>oprI B1 oprL<br>B03 ExoS**	ndh	ndh	ndh	Atpa008<br>Cl-6h<br>188 rru	Atpa010<br>Cl-10h<br>213 rru	ndh	ndh
	ndh	ndh	ndh	Atpa009<br>Cl-10h<br>193 rru	Atpa011<br>Cl-48h	ndh	ndh
**Interpratation**	ndh	ndh	ndh	Synergy:<br>Increased clearing effect timing.	Protocooperation:<br>Clearing effect timing is the same.	ndh	ndh
**PA7<br>261 rru**	Cl-10h<br>246 rru	-<br>PRM<br>298 rru	-<br>212 rru	-<br>PRM<br>283 rru	Cl-14h<br>PRM<br>296 rru	Cl-8h<br>247 rru	Cl-10h<br>PRM<br>297 rru
**MDR<br>O12-serotype<br>TPS<br>ExlA<br>oprI F1 oprL E03**	Atpa001<br>-<br>280 rru	Atpa001<br>-<br>280 rru	Atpa005<br>-<br>256 rru	Atpa008<br>-<br>236 rru	Atpa010<br>-<br>PRM<br>275 rru	Atpa012<br>-<br>PRM<br>287 rru	Qatpa008<br>Cl-20h<br>275 rru
	Atpa002<br>-<br>255 rru	Atpa003<br>284 rru	Atpa006<br>-<br>208 rru	Atpa009<br>-<br>227 rru	Atpa011<br>-<br>Cl-14h	Atpa013<br>-<br>PRM<br>288 rru	Qatpa009<br>-<br>236 rru
	Atpa003<br>-<br>284 rru	Atpa004<br>-<br>265 rru	n/a	n/a	n/a	Atpa014<br>Cl-8h<br>274 rru	Qatpa010<br>Cl-10h<br>297 rru
**Interpratation**	Synergy:<br>clearing effect timing for 10h.	Antagonism or not sufficient pfu/ml:	-	Antagonism or not sufficient pfu/ml:	Protocooperation:	Protocooperation:	Antagonism or not sufficient pfu/ml:

	could be not sufficient pfu/ml.	Increased rru		Increased rru	Clearing effect timing is the same.<br>But with PRM, could be not sufficient pfu/ml.	Clearing effect timing is the same.	Shorten clearing effect timing.

Table S4. Characteristics and interpretation of OmniLog propagation curves for combinations of *K. pneumoniae* strains and individual phages and phage cocktails.

**K. pneumoniae strains/ Capsule type**	**<u>Cocktail 1</u> Phage components**	**<u>Cocktail 2</u> Phage components**	**<u>Cocktail 3</u> Phage components**	**<u>Cocktail 4</u> Phage components**	**<u>Cocktail 5</u> Phage components**	**<u>Cocktail 6</u> Phage components**	**<u>Cocktail 7</u> Phage components**
**0682 192 rru**	Cl-48h	Cl-48h	Cl-48h	Cl-48h	Cl-48h	Cl-48h	Cl-48h 196 rru
**K81**	Atkp001 In-48h 163 rru	Atkp001 In-48h 163 rru	Atkp004 Cl-48h	Atkp007 ln-48h 136 rru	Atkp009 Cl-48h	Atkp012: Cl-48h	Atkp014: Cl-48h
	Atkp008 In-48h 16 rru	Atkp009 In-48h 106 rru	Atkp006 In-48h 180 rru	Atkp008 In-48h 116 rru	Atkp010 ln-48h	Atkp016: Cl-48h	Atkp015: In-48h 112 rru
	n/a	n/a	n/a	n/a	n/a	n/a	Atkp016: Cl-48h
**Interpratation**	<u>Proto-cooperation</u>: Clearing effect timing is the same.	<u>Proto-cooperation</u>: Clearing effect timing is the same.	<u>Proto-cooperation</u>: Clearing effect timing is the same.	<u>Proto-cooperation</u>: Clearing effect timing is the same.	<u>Proto-cooperation</u>: Clearing effect timing is the same.	<u>Proto-cooperation</u>: Clearing effect timing is the same.	<u>Proto-cooperation</u>: Clearing effect timing is the same.
**atcc27736 250 rru**	ndh	IN-8h 178 rru	ndh	ndh	ndh	Cl-48h	Cl-48h
**Serotype-30**	ndh	Atkp001 Cl-0h 250 rru	ndh	ndh	ndh	Atkp012: Cl-48h	Atkp014: Cl-12h 218 rru
	ndh	Atkp009 Cl-0h 250 rru	ndh	ndh	ndh	Atkp016: Cl-48h	Atkp015: Cl-0h
	ndh	n/a	ndh	ndh	ndh	n/a	Atkp016: Cl-48h
**Interpratation**	ndh	<u>Synergy:</u> Increased clearing effect timing.	ndh	ndh	ndh	<u>Proto-cooperation</u>: Clearing effect timing is the same.	<u>Synergy:</u> Increased clearing effect timing.

**nctc13438 272 rru**	ndh	ndh	ndh	ndh	ndh	Cl-48h	Cl-48h
**carbapenemase KPC-3**	ndh	ndh	ndh	ndh	ndh	Atkp012 Cl-0h	Atkp014 Cl-0h
	ndh	ndh	ndh	ndh	ndh	Atkp016 Cl-16h 201 rru	Atkp015 Cl-0h
	ndh	ndh	ndh	ndh	ndh	n/a	Atkp016 Cl-16h 201 rru
**Interpratation**	ndh	ndh	ndh	ndh	ndh	Synergy: Increased clearing effect timing.	Synergy: Increased clearing effect timing.
**SB4385 255 rru**	ndh	ndh	ndh	ndh	ndh	Cl-48h	Cl-48h
**K1**	ndh	ndh	ndh	ndh	ndh	Atkp012 Cl-6h	Atkp014 Cl-0h
	ndh	ndh	ndh	ndh	ndh	Atkp016 Cl-48h	Atkp015 Cl-0h
	ndh	ndh	ndh	ndh	ndh	n/a	Atkp016 Cl-48h
**Interpratation**	ndh	ndh	ndh	ndh	ndh	Proto-cooperation: Clearing effect timing is the same.	Proto-cooperation: Clearing effect timing is the same.
**10394 265 rru**	ndh	ndh	ndh	ndh	ndh	In-48h 206 rru	In-48h 185 rru
**K62**	ndh	ndh	ndh	ndh	ndh	Atkp012 Cl-0h	Atkp014 Cl-0h
	ndh	ndh	ndh	ndh	ndh	Atkp016 Cl-0h	Atkp015 Cl-0h
	ndh	ndh	ndh	ndh	ndh	n/a	Atkp016 Cl-0h
	ndh	ndh	ndh	ndh	ndh	Synergy: Inhibition effect up to 48h	Synergy: Inhibition effect up to 48h
**70165**	ndh	ln-48h 194 rru	ln-48h 202 rru	Cl-10 h ln-48h 216 rru	In-48h 185 rru	ndh	ndh
**K2**	ndh	Atkp001 Cl-0h 224 rru	Atkp004: ln-48h 201 rru	Atkp007 Cl-8h 257 rru	Atkp010 In-48h 185 rru	ndh	ndh
	ndh	Atkp009 Cl-0h 246 rru	Atkp006: Cl-0h 246 rru	Atkp008 Cl-8h 243 rru	Atkp009 Cl-0h 246 rru	ndh	ndh

**Interpratation**	ndh	Synergy: Increases clearing effect timing.	Proto-cooperation: Clearing effect timing is the same.	Synergy: Increases clearing effect timing.	Proto-cooperation: Clearing effect timing is the same.	ndh	ndh
**VPKP389**	Cl-48h	Cl-28h In-48h	ndh	ndh	Cl-48h	ndh	ndh
**K27**	Atkp001 Cl-0h	Atkp001 Cl-0h	ndh	ndh	Atkp009 In-48h	ndh	ndh
	Atkp008 Cl-12h	Atkp009 In-48h	ndh	ndh	Atkp010 Cl-0h	ndh	ndh
**Interpratation**	Synergy: Increases clearing effect timing.	Proto-cooperation: Clearing effect timing is the same.	ndh	ndh	Proto-cooperation: Clearing effect timing is the same.	ndh	ndh

Abbreviations: CL, clearing; In, Inhibition; rru, relative respiration unit; PRM, phage resistant mutant; ndh, not described here; "-", no activity; n/a, not applicable.

Figures S1-S15. P. aeruginosa phages EOP.

Figure S1. EOP of P. aeruginosa phage Atpa001

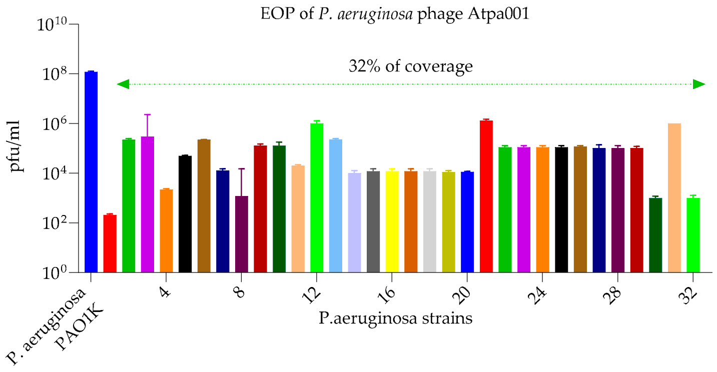

Figure S2. EOP of P. aeruginosa phage Atpa002

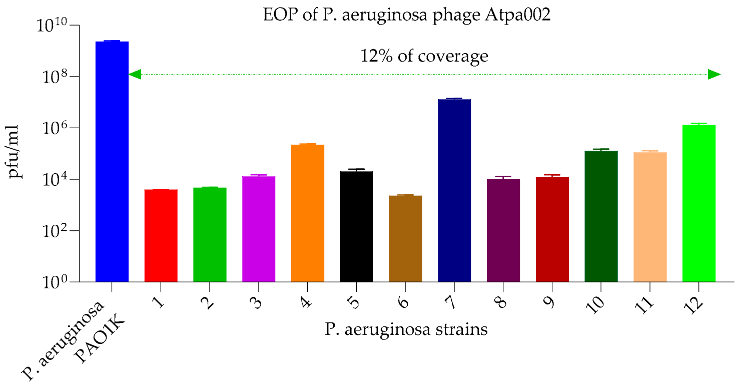

.

Figure S3. EOP of P. aeruginosa phage Atpa003

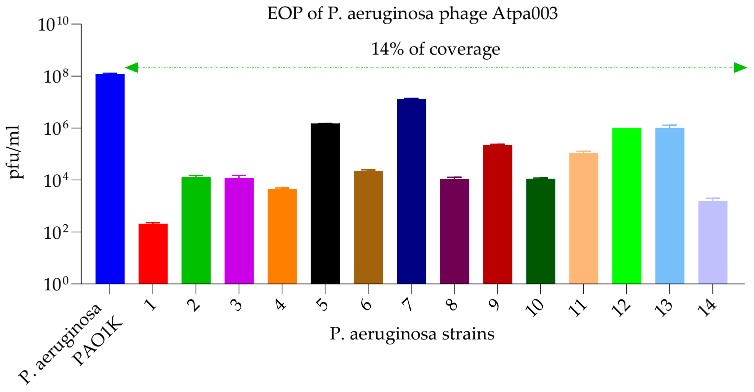

Figure S4. EOP of P. aeruginosa phage Atpa004

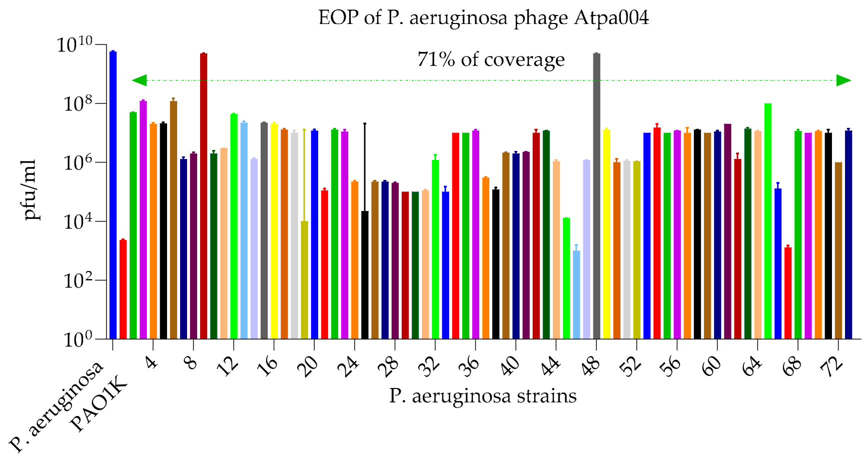

Figure S5. EOP of P. aeruginosa phage Atpa005

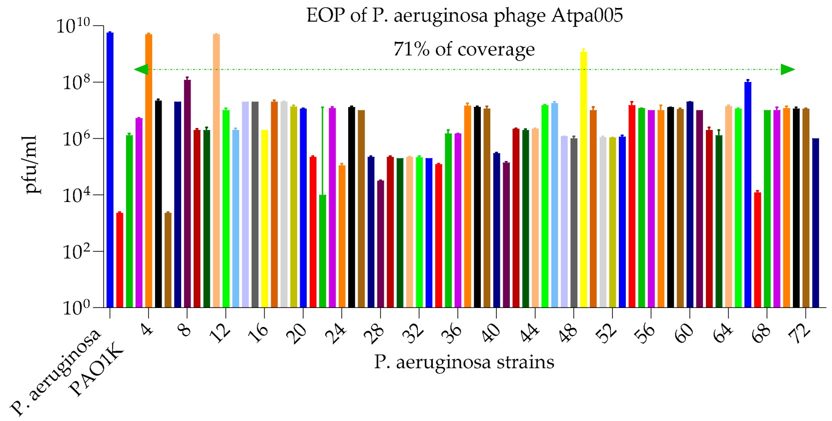

Figure S6. EOP of P. aeruginosa phage Atpa006.

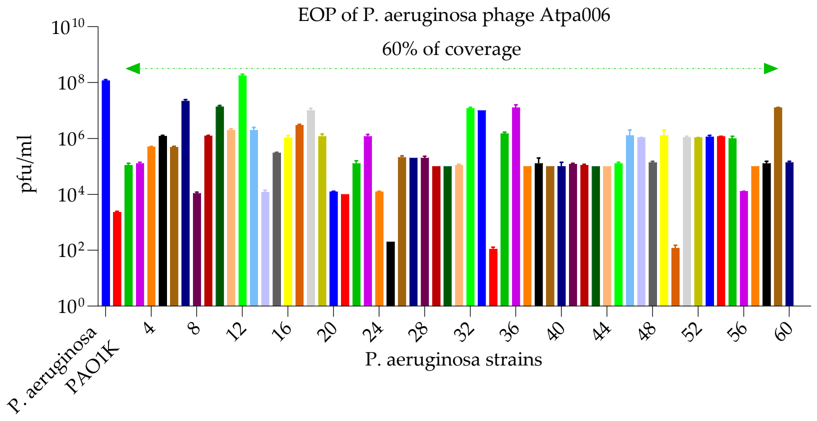

Figure S7. EOP of P. aeruginosa phage Atpa008

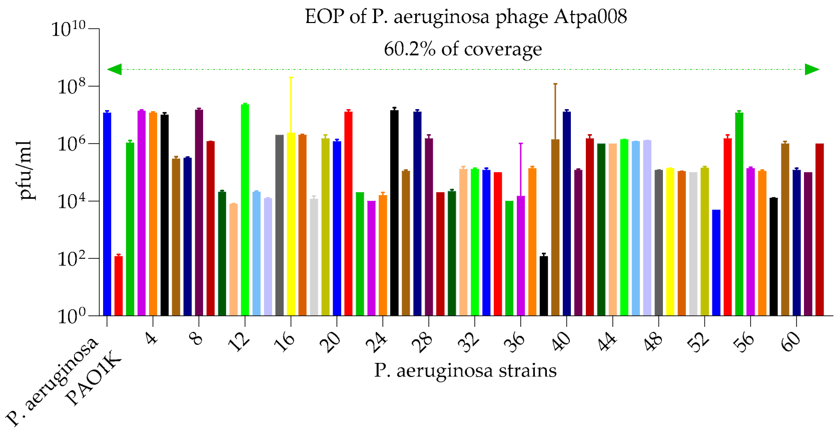

Figure S8. EOP of P. aeruginosa phage Atpa009.

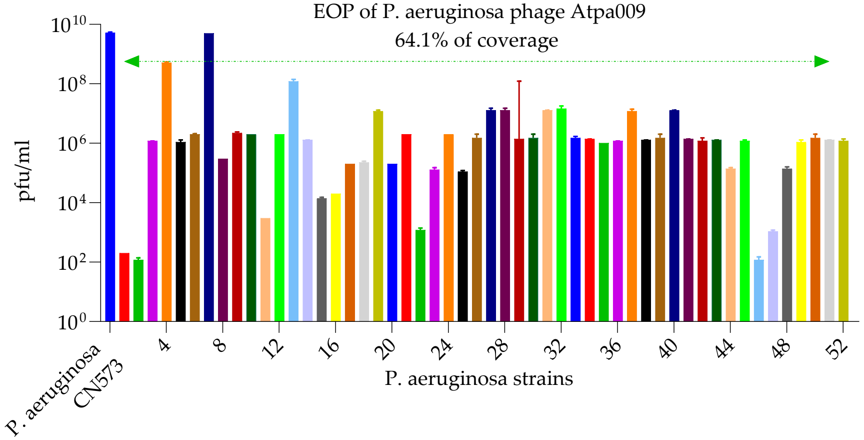

Figure S9. EOP of P. aeruginosa phage Atpa010

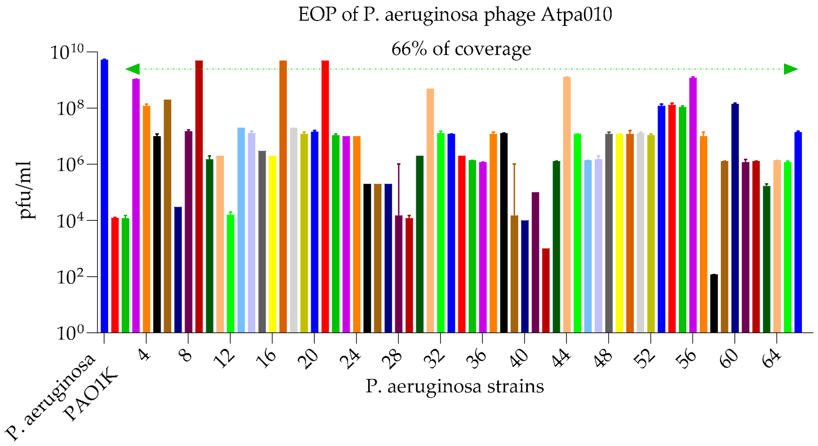

Figure S10. EOP of P. aeruginosa phage Atpa011

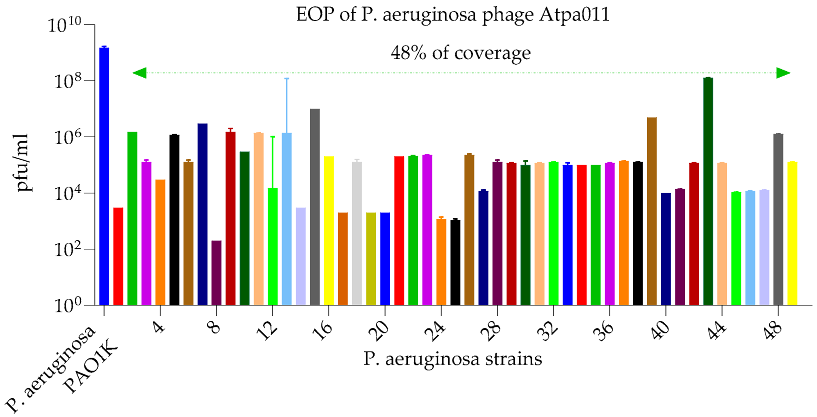

Figure S11. EOP of P. aeruginosa phage Atpa012

Figure S12. EOP of P. aeruginosa phage Atpa013

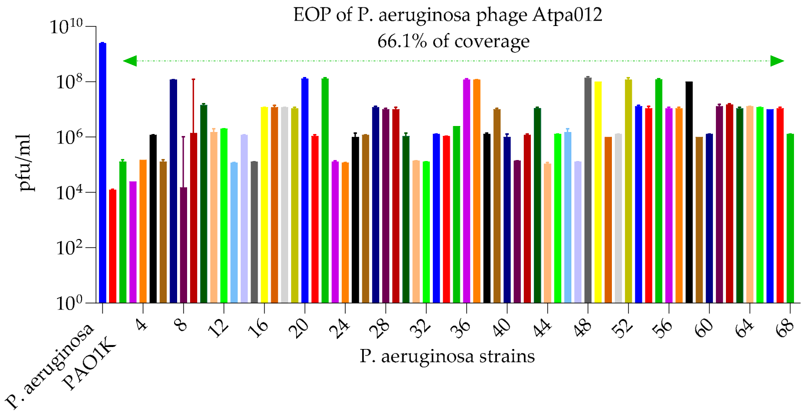

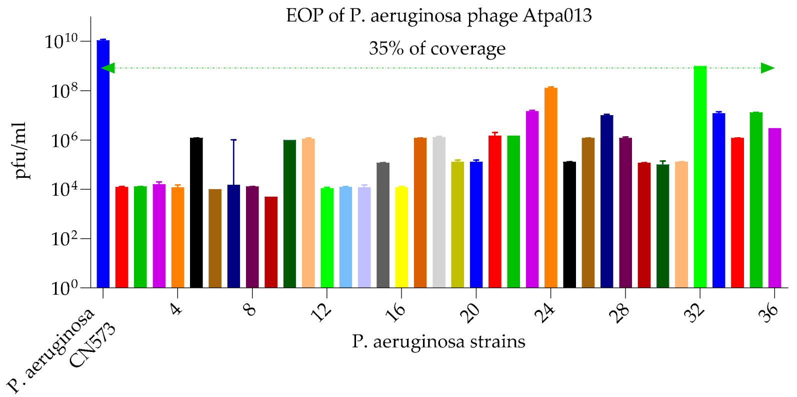

Figure S13. EOP of P. aeruginosa phage AQapa008

Figure S14. EOP of P. aeruginosa phage AQapa009

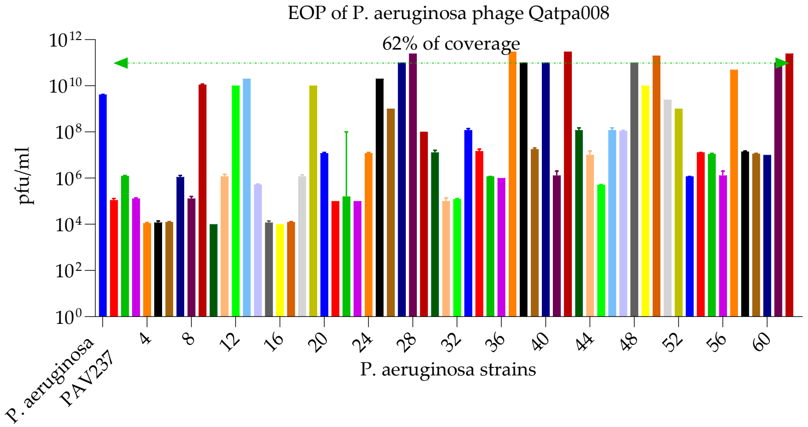

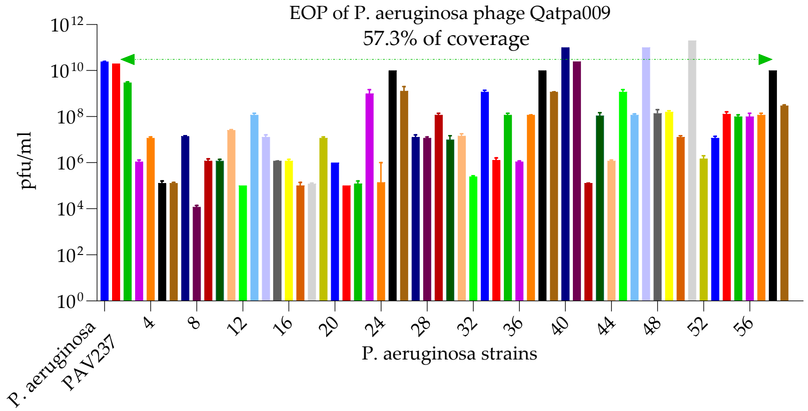

Figure S15. EOP of P. aeruginosa phage AQapa010

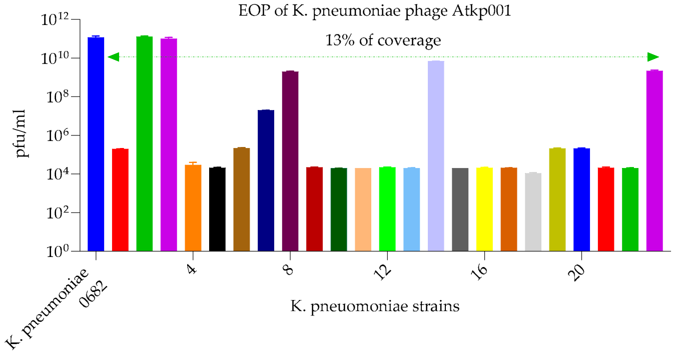

Figures S16-S25. K. pneumoniae phages EOP.

Figure S16. EOP of K. pneumoniae phage Atkp001

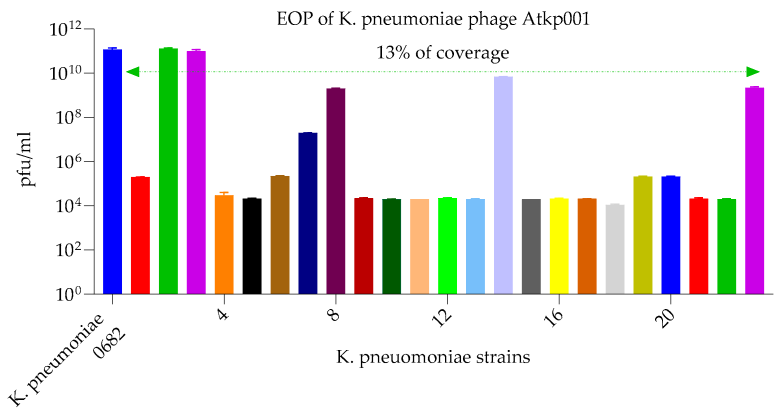

Figure S17. EOP of K. pneumoniae phage Atkp004

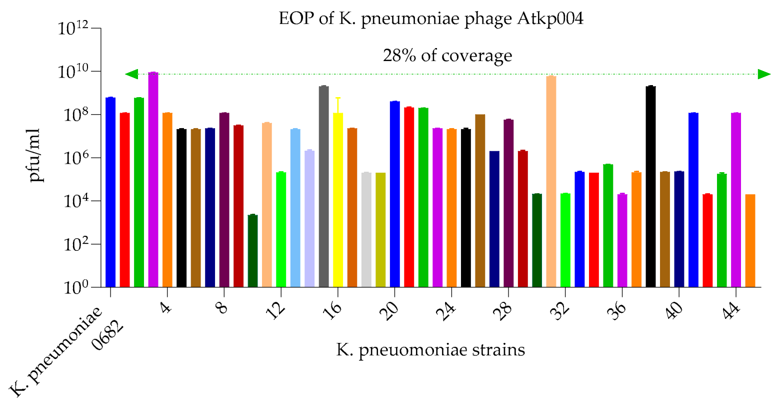

Figure S18. EOP of K. pneumoniae phage Atkp006

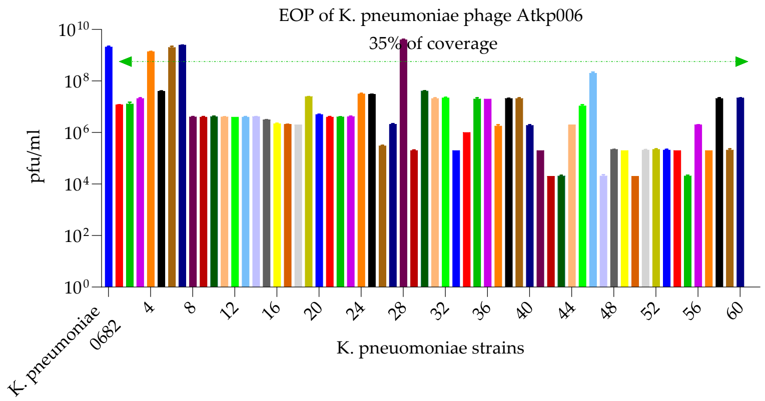

Figure S19. EOP of K. pneumoniae phage Atkp007

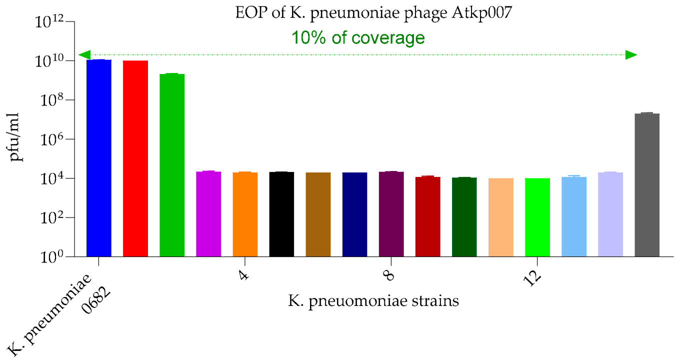

Figure S20. EOP of K. pneumoniae phage Atkp008

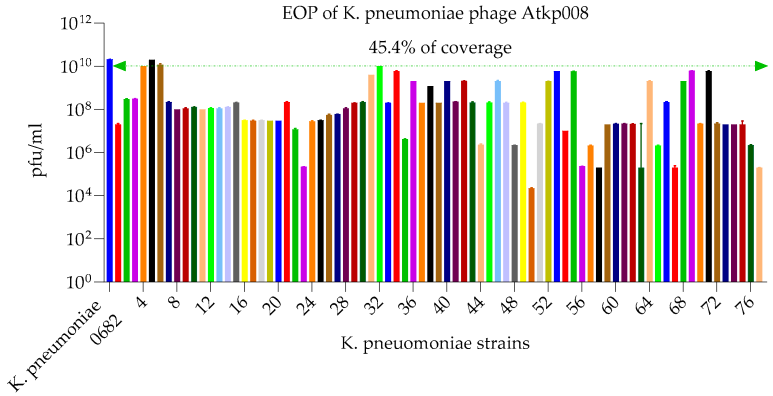

Figure S21. EOP of K. pneumoniae phage Atkp009

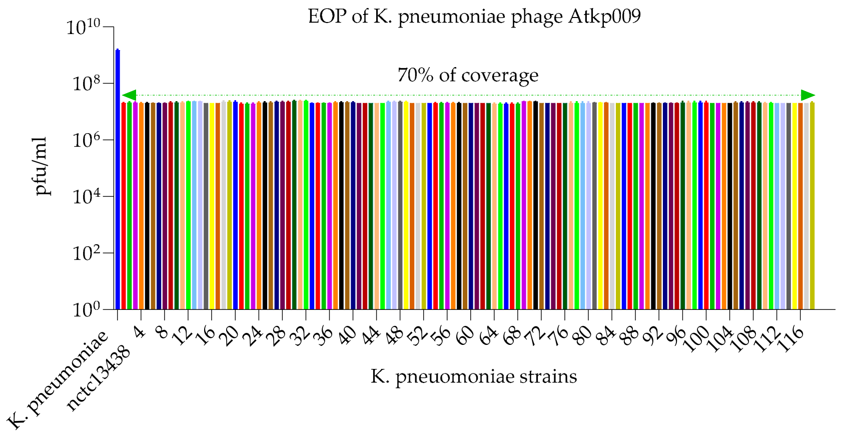

Figure S22. EOP of K. pneumoniae phage Atkp012

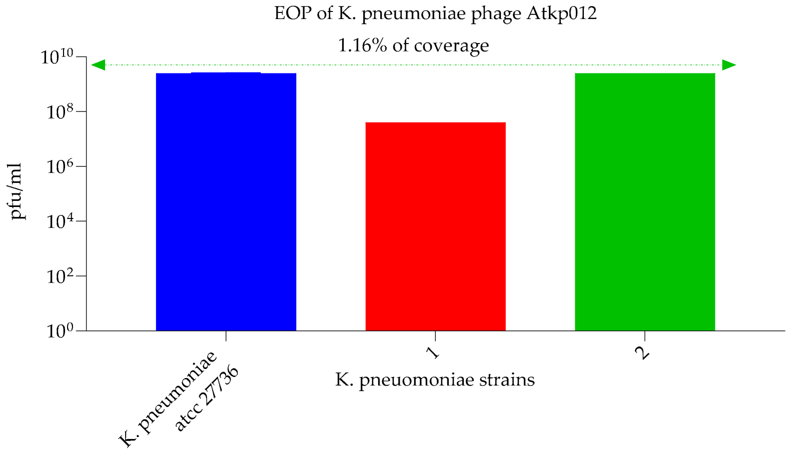

Figure S23. EOP of K. pneumoniae phage Atkp014

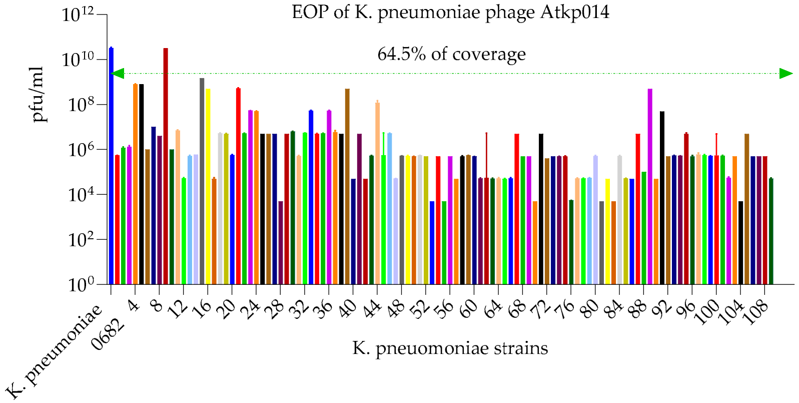

Figure S24. EOP of K. pneumoniae phage Atkp015

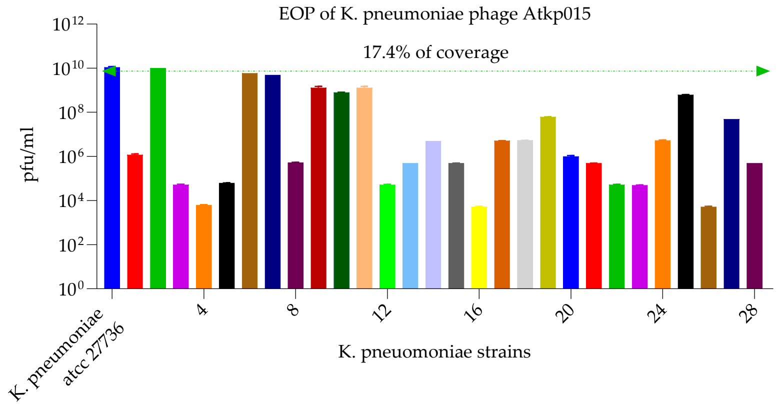

Figure S25. EOP of K. pneumoniae phage Atkp016

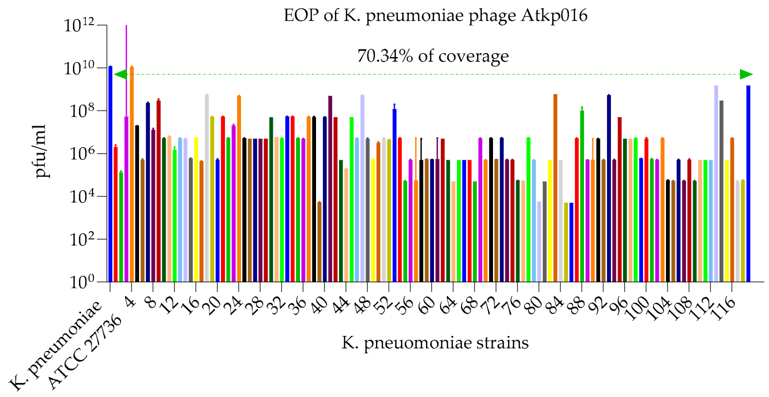

Figures S26-S40. Appelmans assay of P. aeruginosa phages.

Figure S26. Appelmans assay of P. aeruginosa phage Atpa001

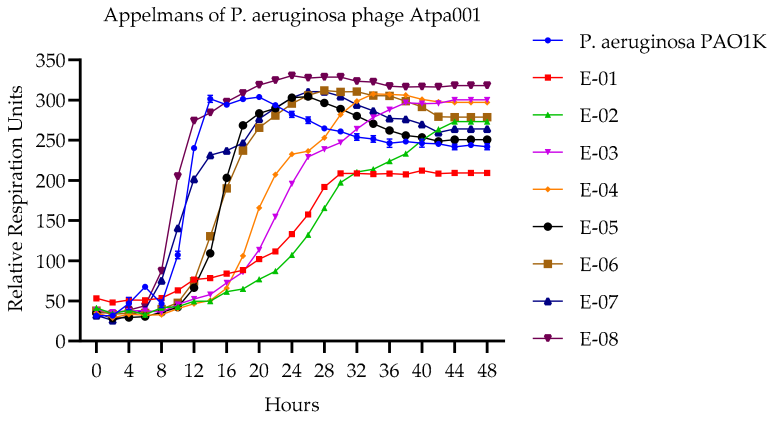

Figure S27. Appelmans assay of P. aeruginosa phage Atpa002

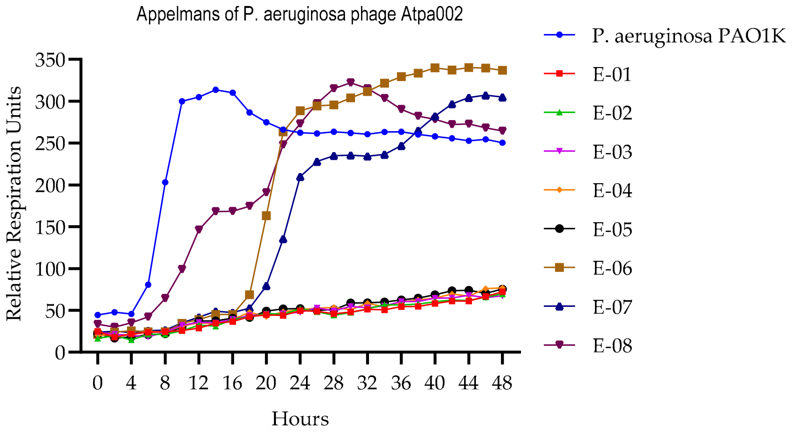

Figure S28. Appelmans assay of P. aeruginosa phage Atpa003

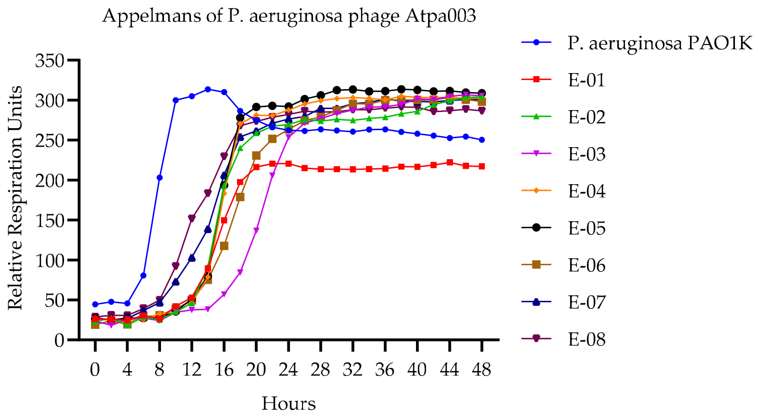

Figure S29. Appelmans assay of P. aeruginosa phage Atpa004

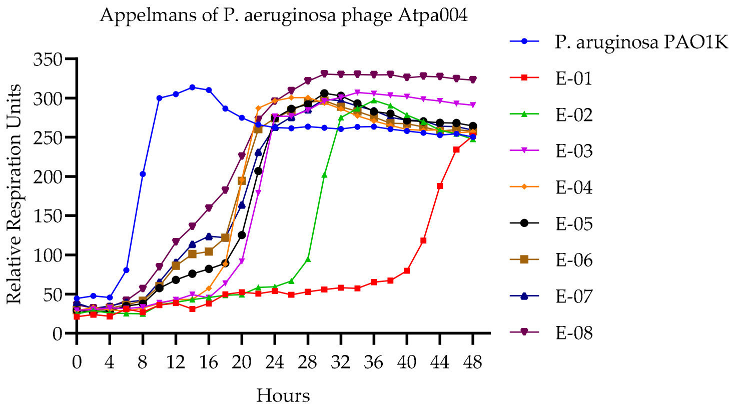

Figure S30. Appelmans assay of P. aeruginosa phage Atpa005

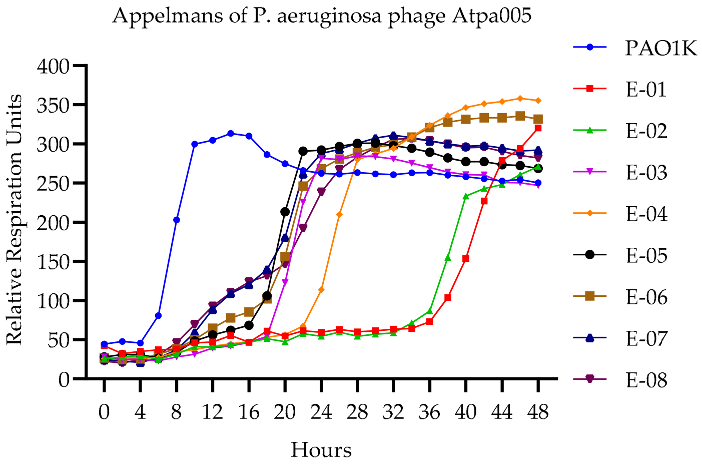

Figure S31. Appelmans assay of P. aeruginosa phage Atpa006

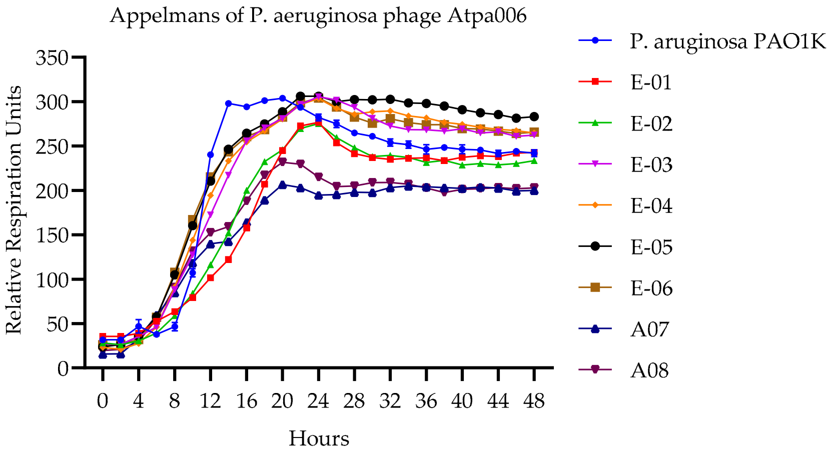

Figure S32. Appelmans assay of P. aeruginosa phage Atpa008

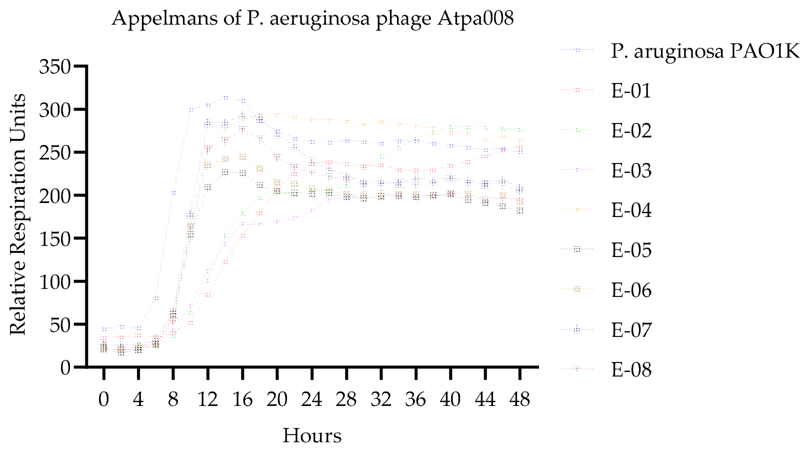

Figure S33. Appelmans assay of P. aeruginosa phage Atpa009

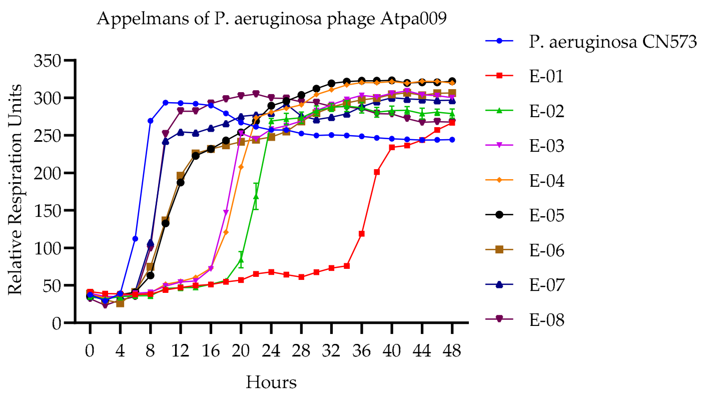

Figure S34. Appelmans assay of P. aeruginosa phage Atpa010

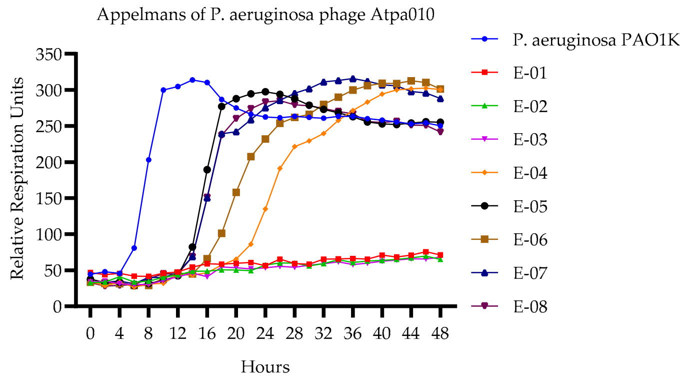

Figure S35. Appelmans assay of P. aeruginosa phage Atpa011

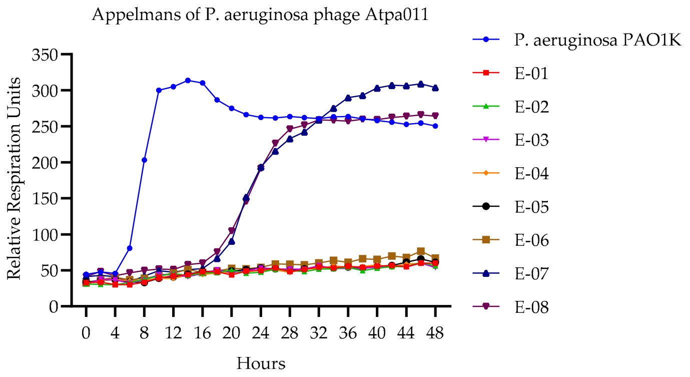

Figure S36. Appelmans assay of P. aeruginosa phage Atpa012

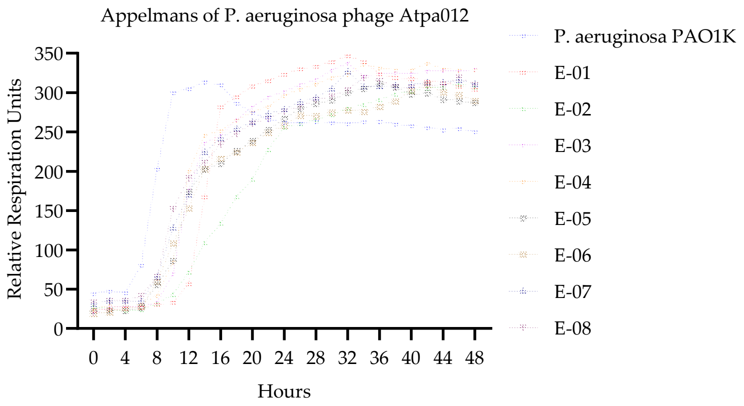

Figure S37. Appelmans assay of P. aeruginosa phage Atpa013

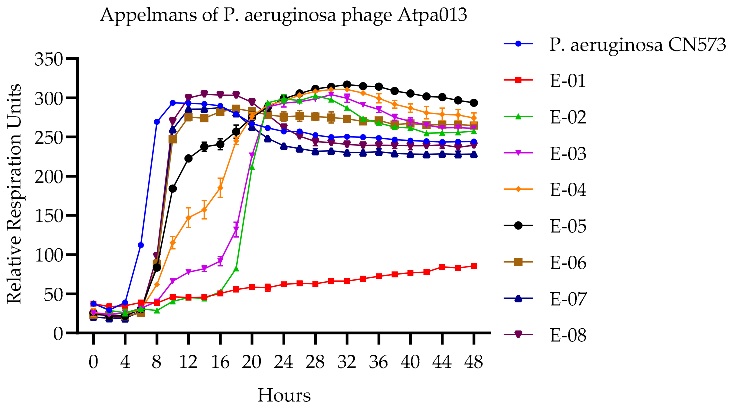

Figure S38. Appelmans assay of P. aeruginosa phage Qatpa008

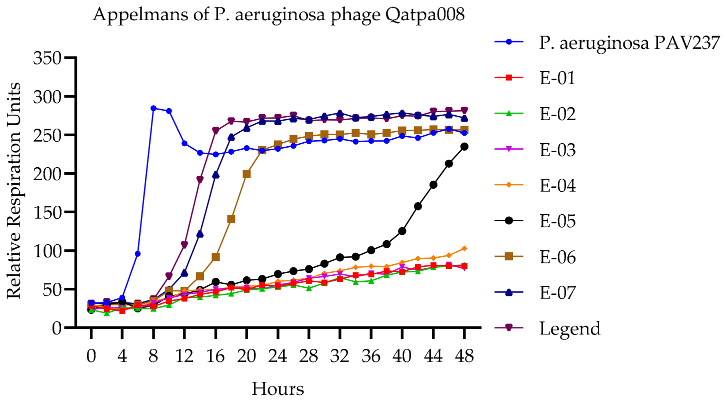

Figure S39. Appelmans assay of P. aeruginosa phage Qatpa009

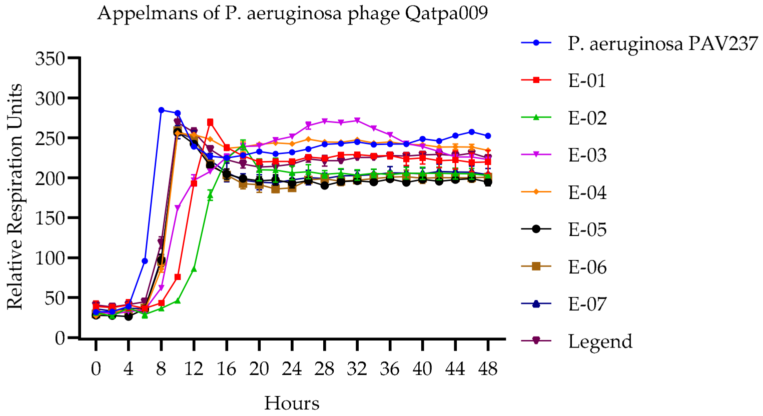

Figure S40. Appelmans assay of P. aeruginosa phage Qatpa010

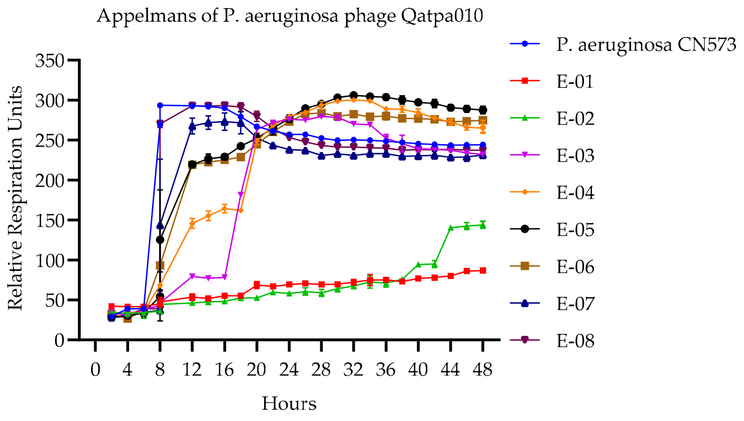

Figures S41-S50. Appelmans assay of K. pneumoniae phages.

Figure S41. Appelmans assay of P. aeruginosa phage Atkp001

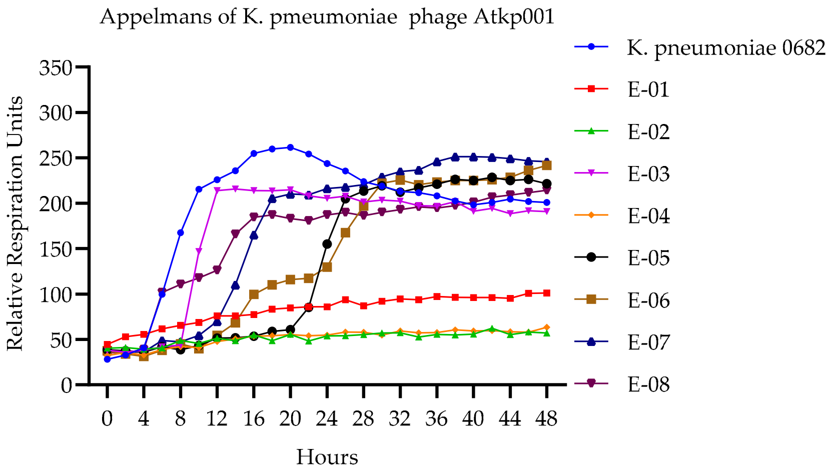

Figure S42. Appelmans assay of P. aeruginosa phage Atkp004

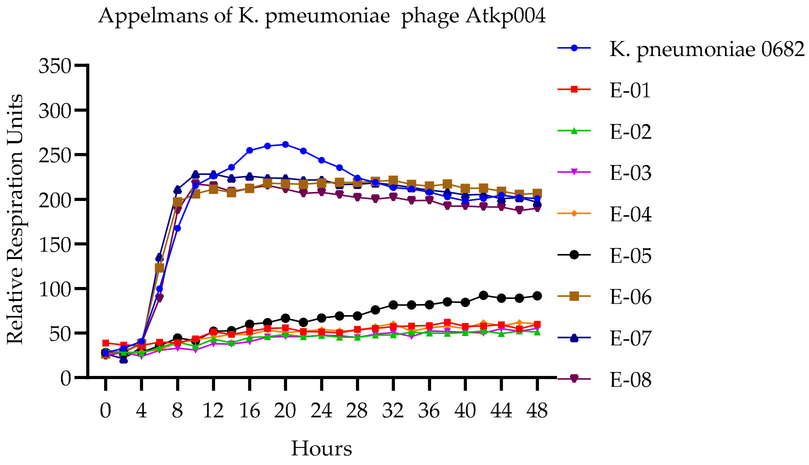

Figure S43. Appelmans assay of P. aeruginosa phage Atkp006

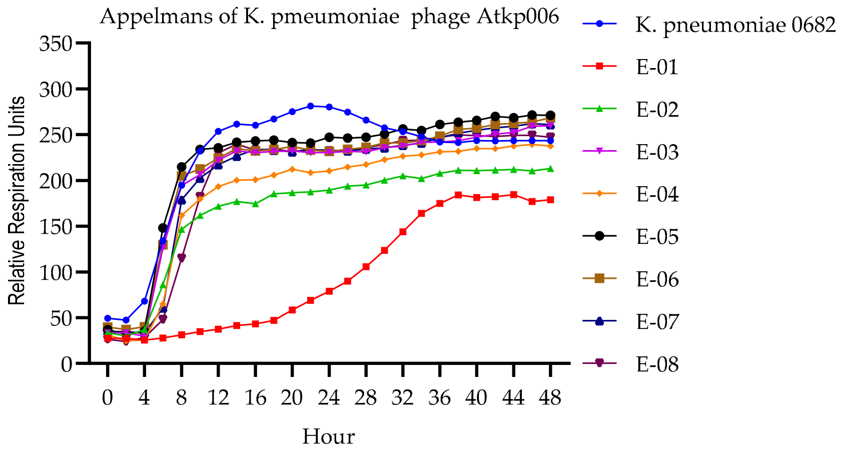

Figure S44. Appelmans assay of P. aeruginosa phage Atkp007

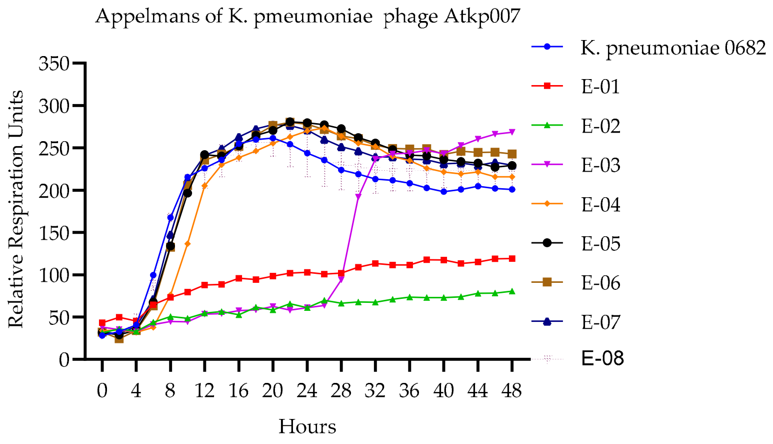

Figure S45. Appelmans assay of P. aeruginosa phage Atkp008

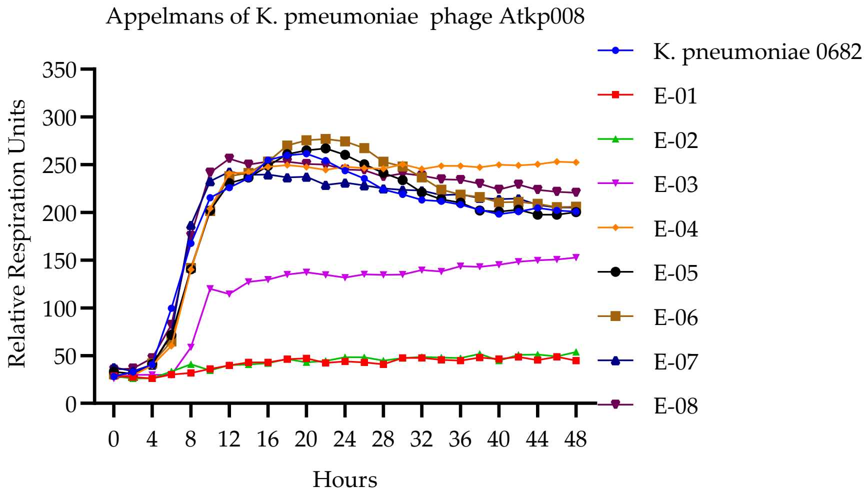

Figure S46. Appelmans assay of P. aeruginosa phage Atkp009

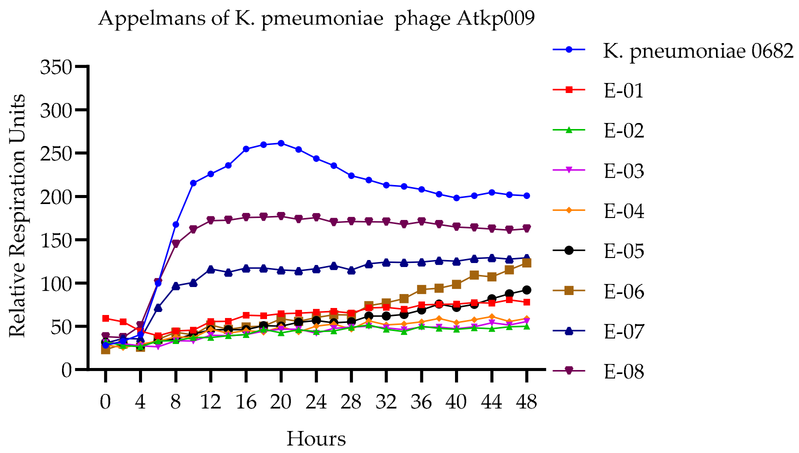

Figure S47. Appelmans assay of P. aeruginosa phage Atkp010

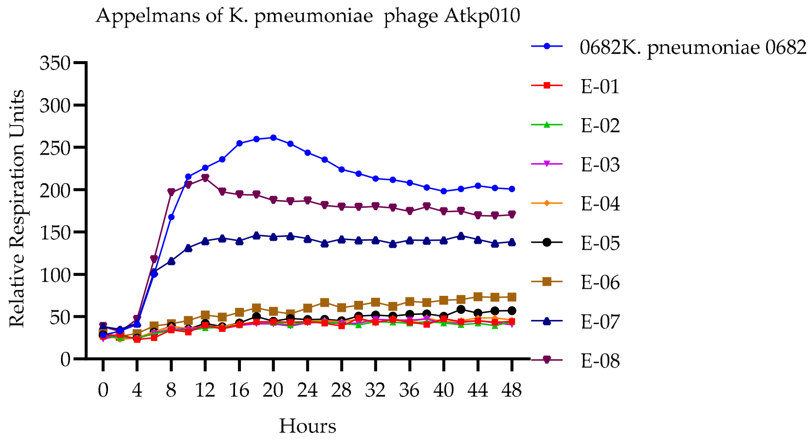

Figure S48. Appelmans assay of P. aeruginosa phage Atkp012

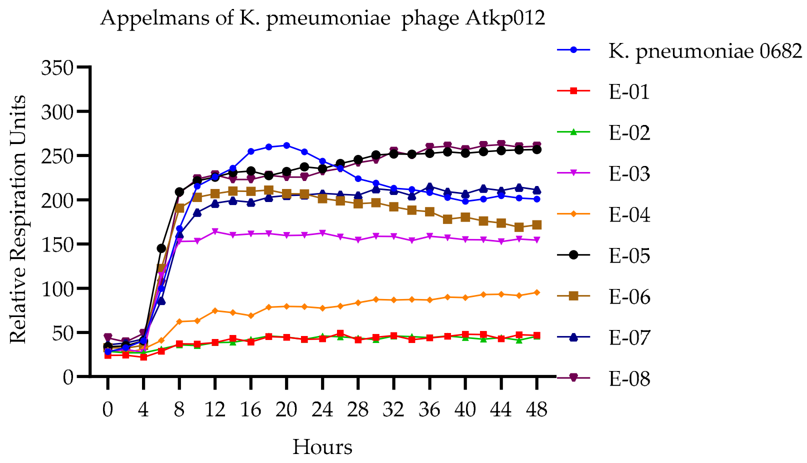

Figure S49. Appelmans assay of P. aeruginosa phage Atkp014

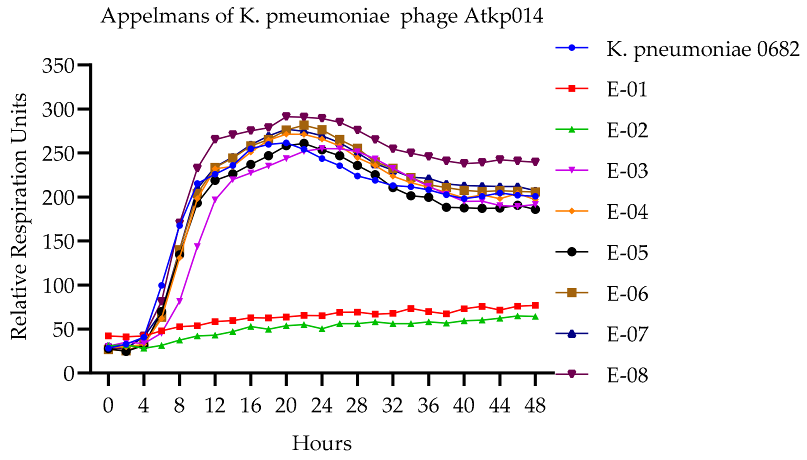

Figure S50. Appelmans assay of P. aeruginosa phage Atkp015

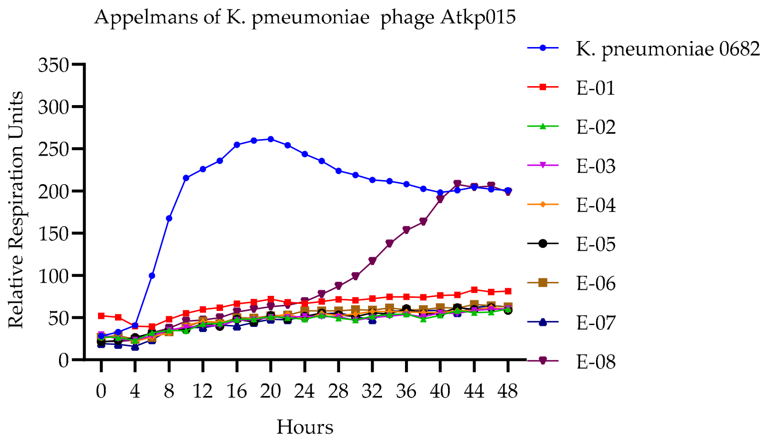

Figure S51. The determination of cfu/ml and pfu/ml at the end point of the OmniLog 48 h incubation of P. aeruginosa phage cocktails on CN573.

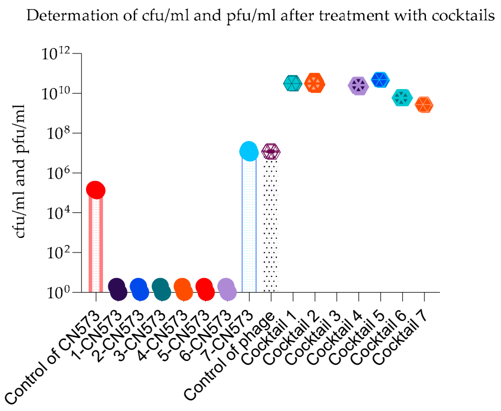

Figure S52. The determination of cfu/ml and pfu/ml at the end point of the OmniLog 48 h incubation of P. aeruginosa phage cocktails on PAV237.

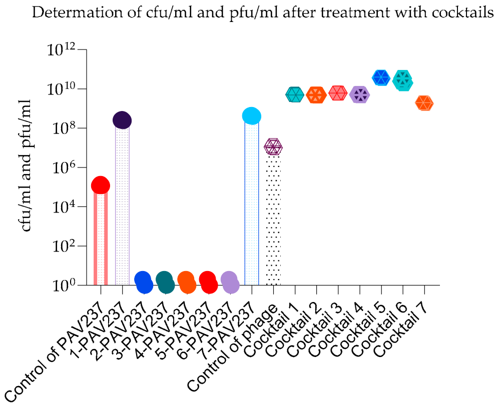

Figure S53. (a) the lytic activity curves of P. aeruginosa phage's Atpa001-Atpa009 and cocktails 1-4 on PAV237; (b) the lytic activity curves of P. aeruginosa phage's Atpa009-Atpa014, Qatpa008-Qatpa010 and cocktails 5-7 on PAV237.

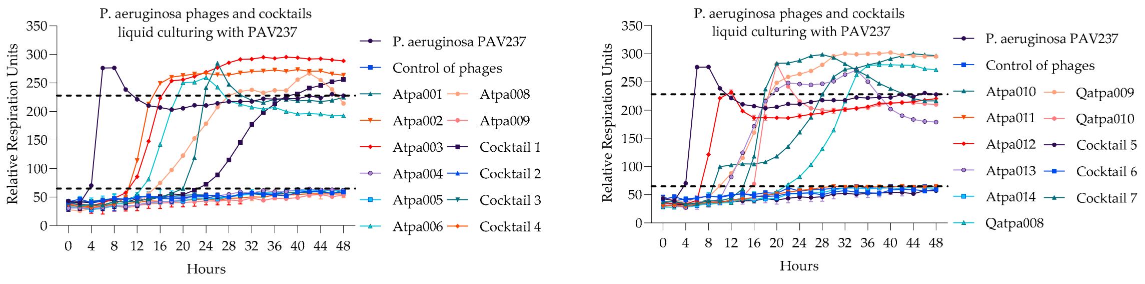

(**a**) (**b**)

Figure S54. (a) the lytic activity curves of P. aeruginosa phage's Atpa001-Atpa006 and cocktails 1, 3 on PAO1; (b) the lytic activity curves of P. aeruginosa phage's Atpa005-Atpa006 and Atpa010- Atpa011 and cocktails 3, 5 on PA14.

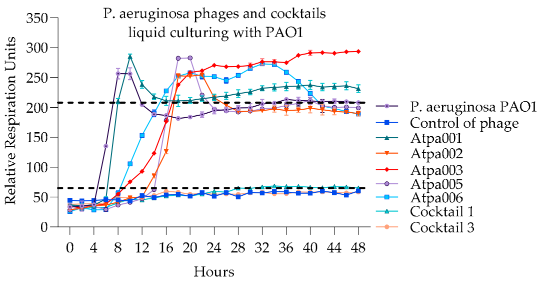

(**a**)

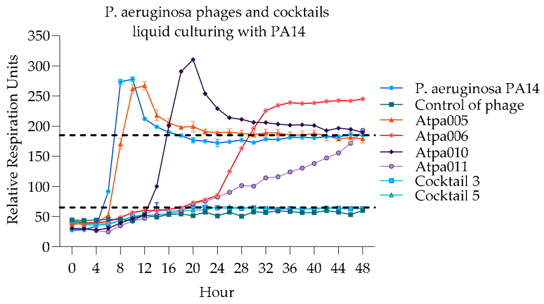

(**b**)

Figure S55. (a) the lytic activity curves of P. aeruginosa phage's Atpa001-Atpa003 and cocktails 1, 5 on A11; (b) the lytic activity curves of P. aeruginosa phage's Atpa012-Atpa014, Qatpa008-Qatpa010 and cocktails 6-7 on Is573.

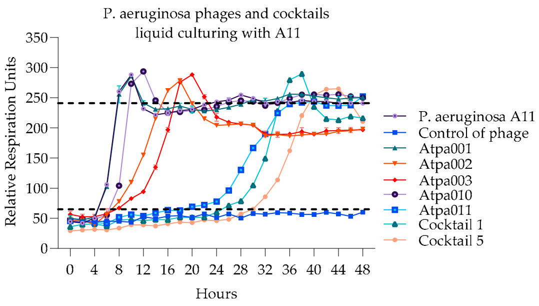

(**a**)

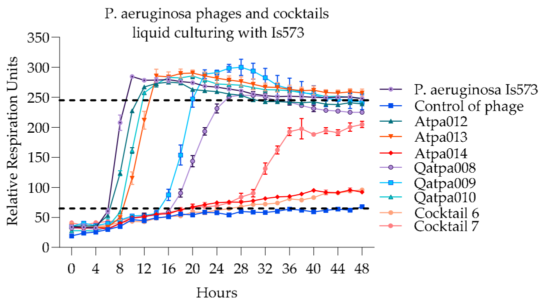

(**b**)

Figure S56. (a) the lytic activity curves of P. aeruginosa phage's Atpa008-Atpa011 and cocktails 4-5 on Is580; (b) the lytic activity curves of P. aeruginosa phage's Atpa001-Atpa009 and cocktails 1-4 cocktails 5-7 on PA7.

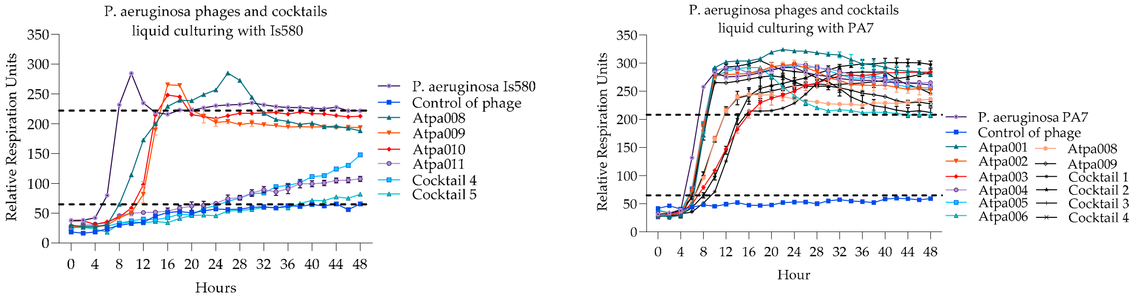

(**a**) (**b**)

Figure S57. (a) the lytic activity curves of P. aeruginosa phage's Atpa009-Atpa014, Qatpa008-Qatpa010 and cocktails 6-7 on PA7 ; (b) the lytic activity curves of K. pneumoniae phage's Atkp012-Atkp016 and cocktails 6-7 on nctc13438 curves.

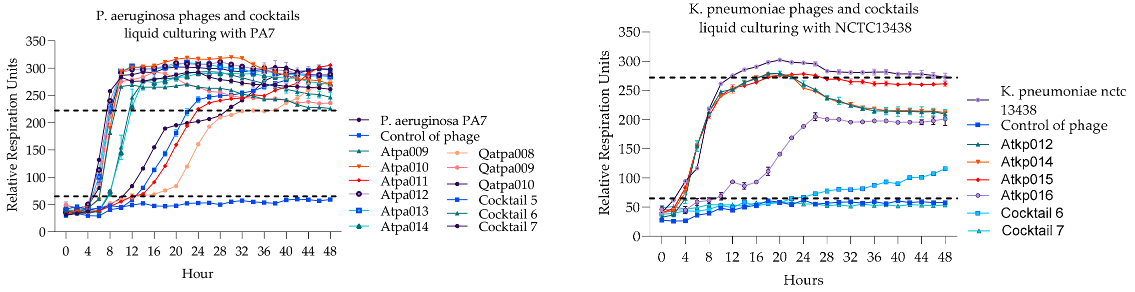

(**a**) (**b**)

Figure S58. (a) the lytic activity curves of P. aeruginosa phage's Atpa009-Atpa014, Qatpa008-Qatpa010 and cocktails 6-7 on PA7 ; (b) the lytic activity curves of K. pneumoniae phage's Atkp012-Atkp016 and cocktails 6-7 on nctc13438 curves.

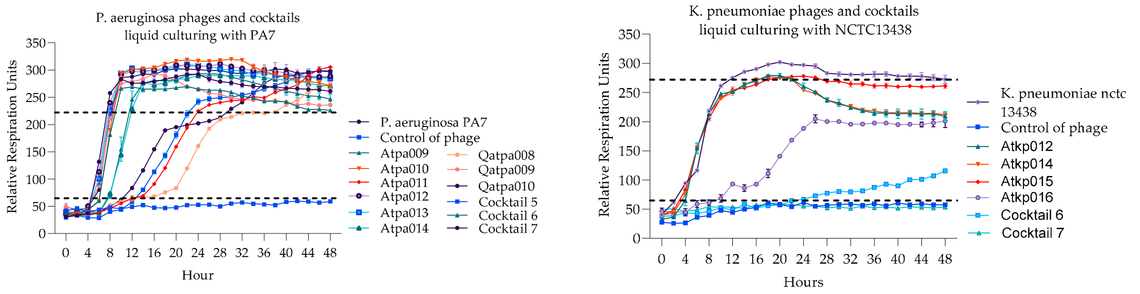

(**a**) (**b**)

Figure S59. (a) the lytic activity curves of K. pneumoniae Atkp012-Atkp016 and cocktails 6-7 on SB4385; (b) the lytic activity curves of K. pneumoniae phage's Atkp012-Atkp016 and cocktails 6-7 on 10394 curves.

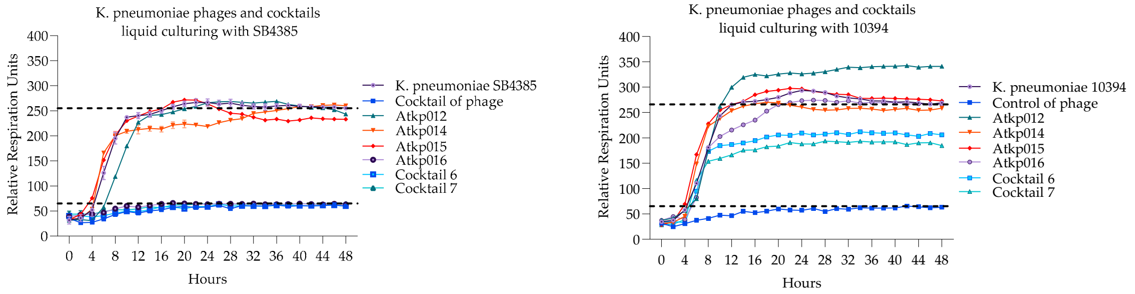

(**a**) (**b**)

Figure S60. (a) the lytic activity curves of K. pneumoniae Atkp012-Atkp016 and cocktails 6-7 on VKPKP389. (b) the lytic activity curves of K. pneumoniae phage's Atkp001-Atkp009 and cocktails 1-4 on 70165.

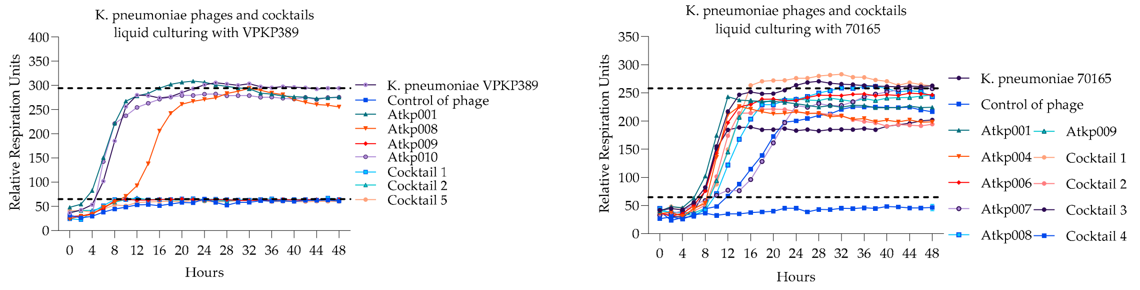

(**a**) (**b**)
